# Density- and Temperature-Dependent Potentials: Redefinition of the Local Density to Improve the Simulation of Liquids within Generalized Dissipative Particle Dynamics

**DOI:** 10.1021/acs.jctc.5c01646

**Published:** 2025-12-16

**Authors:** Giuseppe Colella, James P. Larentzos, Fernando Bresme, Josep Bonet Avalos

**Affiliations:** † Departament d’Enginyeria Química, ETSEQ, 16777 Universitat Rovira i Virgili, Tarragona 43007, Spain; ‡ U.S. Army Combat Capabilities Development Command (DEVCOM) Army Research Laboratory, Aberdeen Proving Ground, Maryland 21005, United States; § Department of Chemistry, Molecular Sciences Research Hub, 4615 Imperial College London, London W12 0BZ, U.K. and Thomas Young Centre for Theory and Simulation of Materials, Imperial College London, London SW7 2AZ, U.K.

## Abstract

In this work, we apply the mesoscopic Generalized Energy-Conserving Dissipative Particle Dynamics (GenDPDE) method (J. Bonet Avalos et al., *Phys. Chem. Chem. Phys.* **2019**, 21, 24891) to analyze liquid phases. We demonstrate that the traditional DPD estimator of the particle local density is inadequate for the simulation of liquid phase conditions, as it leads to an unphysical behavior, typically in the form of particle clustering, that modifies the local structure and prevents the system from equilibrating at the sought thermodynamic state point. We therefore propose an alternative definition for the local density calculation which significantly improves its estimation and crucially allows us to recover physically meaningful results. We prove the beneficial effects of this redefinition by analyzing the thermodynamic properties and local structure of liquid argon and water, drawing a comparison between the outcome of equilibrium simulations considering the two density estimators. With the introduction of the new local density expression, GenDPDE is suitable for the qualitative and quantitative analysis of complex fluid systems in a variety of relevant scenarios, including liquid phases.

## Graphical abstract

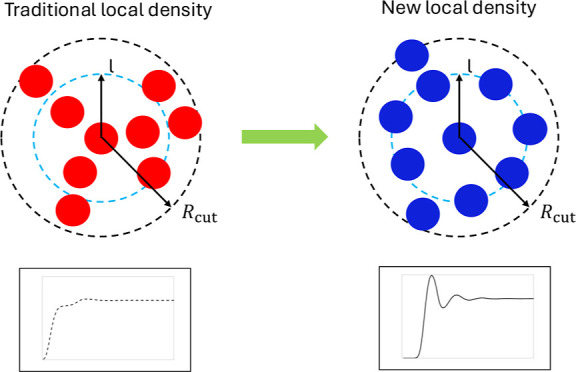

## 1 Introduction

In recent decades, there has been an increasing interest in mesoscopic approaches to the numerical analysis of complex systems, such as polymer solutions,^1–3^ fluid–solid interfaces,^4,5^ liquid-crystalline systems,^6^ colloidal suspensions,^7^ among many others. These systems, which involve a high number of atoms and molecular groups, are characterized by time and length scales that are typically large in comparison with the scales of the molecular constituents. Thus, the application of atomistic techniques, such as molecular dynamics (MD), to the study of these complex fluids is often computationally prohibitive. On the other hand, macroscopic computational fluid dynamics tools (CFD) are unable to capture physicochemical processes that occur at the micro- and mesoscale and that often play a crucial role in determining the properties and behavior of such systems. Mesoscopic models have been introduced as an attempt to overcome these issues and bridge the gap between molecular and continuum approaches. Dissipative Particle Dynamics (DPD)^8,9^ is a popular example of this class of models.

DPD is a particle-based, Lagrangian, coarse-grain (CG) method, whose dynamics by construction conserves both the number of particles and the total momentum of the system. Consequently, the continuity equation (related to the particle number density field) and Navier–Stokes equation (related to the momentum density field), and thus the hydrodynamic behavior of the system, are recovered in the hydrodynamic limit of long times and long wavelengths.^10,11^ The dynamics of a DPD system is described by a set of equations of motion (EoM) in which the total force acting on the DPD particle (which we will often refer to as *mesoparticle*) is modeled as the sum of conservative and dissipative contributions. The latter arises as a result of the CG process and accounts for the coupling between the explicitly *resolved* and the remaining *unresolved* degrees of freedom (DoF) of the original underlying physical system. A stochastic contribution is also added to describe the thermal agitation associated with the energy stored in the unresolved DoF, which randomly affects the resolved DoF. This contribution is modeled as a Langevin-like force, whose amplitude is related to the strength of the dissipative contribution through a Fluctuation–Dissipation (FD) theorem.^12^

Albeit suitable for the analysis of many processes of interest,^13–15^ DPD presents several limitations. A fundamental problem lies in its inability to reproduce liquid–vapor coexistence, due to the form of the equation of state (EoS), quadratic in density, associated with the original model.^9^ To tackle this issue, many-body extensions of DPD (MDPD) have been introduced,^16–21^ in which the system’s potential energy depends on many-body, rather than pairwise particle interactions, through the definition of a *local density*. Within this context, more complex EoS can be modeled that include liquid–vapor phase transitions and coexistence. Both DPD and MDPD are nevertheless limited to isothermal conditions, since their dynamics does not conserve total energy, due to the effect of the dissipative forces. Hence, these methods are not suitable for the analysis of scenarios involving, e.g., heat fluxes.

DPD with energy conservation (DPDE)^22,23^ and its later generalization GenDPDE^24,25^ have been developed to overcome this constraint. GenDPDE introduces the concept of the mesoparticle as a property carrier, with an *internal* state characterized by a thermodynamic description. In its simpler version, such an internal state is described through a *local entropy* function that depends on both the local density, related to the volume occupied by the mesoparticle, and the internal energy content, associated with the nonresolved DoF. As a consequence, density- and temperature-dependent potentials can consistently be defined within this framework, making GenDPDE applicable to complex nonequilibrium phenomena involving, e.g., chemical reactions^26^ and shock compression,^27^ among others.

GenDPDE is also capable of reproducing liquid–vapor coexistence, although it has been shown to underestimate the coexistence densities, due to the approximation made in assigning the particle volume from the evaluation of the local weighted density.^24^ An accurate volume calculation is however essential when the target is to simulate liquid phase, as it ensures the correct estimation of the potential contributions to the total energy of the system, since in liquids the potential contributions dominate over the kinetic ones.

In this work, we present a novel estimation of the particle volume, which corrects the deficiencies of the conventional DPD weighted density estimator.^16,17,21^ We show that, when considering liquid conditions, this DPD estimator induces particle clustering that leads to an alteration of the local structure of the system and prevents it from equilibrating at the desired thermodynamic state point. We observe that, in a system where the interparticle forces dominate over thermal agitation, particles can rearrange so as to minimize a global potential energy, exploiting the particular shape of the weighting function used to calculate the local density. Such problems have been overlooked in most applications, where the thermal agitation is comparable or dominant, but they are crucial in the investigation of liquids. We demonstrate that such artifacts can be eliminated if the thermodynamic density is estimated through the alternative volume definition proposed in this work. The main result is therefore the demonstration that physically meaningful analyses of liquids can be attained using GenDPDE, provided that the local thermodynamic density of the system is properly evaluated.

The manuscript is organized as follows. In Section 2, we introduce the general GenDPDE framework, with a particular focus on the local density. We elaborate upon the traditional and alternative expressions for its evaluation, thoroughly discussing the derivation of the latter. In Section 3, we review the GenDPDE algorithm and define the internal thermodynamic model for the mesoparticles used in this work, based on the modified Tait EoS for weakly compressible fluids.^28^ We also provide here the computational details of our numerical simulations, whose outcome is presented in Section 4. In this discussion, liquid argon and water have been considered as test cases. We analyze and compare the results obtained with the two density estimators, examining differences in both the system thermodynamic quantities and local structure and showing the benefits of the proposed alternative estimator. We conclude with a summary of our findings in Section 5.

## 2 Theoretical Framework

In this section, we first provide a concise presentation of the GenDPDE local thermodynamic (LTh) framework, along the lines of refs.,^24,25^ where a more detailed discussion can be found. Then, we expand on the evaluation of the local density through both the traditional DPD estimator and the alternative proposed expression. We highlight the limitations of the traditional estimator in the case of locally structured systems, and discuss how the novel definition allows us to address these deficiencies from a theoretical perspective.

### 2.1 Internal Thermodynamic Description of the Mesoparticle

In the general GenDPDE framework, mesoparticles are regarded as embedding clusters of physical particles whose explicit dynamics has been smoothed out in the CG process. The internal state of the mesoparticle *i* is thus characterized by a set of *surviving* variables, related to the underlying group of physical entities, namely, its center-of-mass position **r**_*i*_, total momentum **p**_*i*_, and total mass *m*_*i*_. Moreover, an internal energy content *u*_*i*_ is considered to be stored in the unresolved DoF of the mesoparticle. Finally, each mesoparticle is also characterized by a volume $V_{i}$ it occupies, or equivalently by a local number density from which the volume is defined according to $n_{i}\equiv 1/V_{i}$ . The local density is a function of the positions of the neighboring mesoparticles and therefore depends on the local environment. With the internal state being defined, GenDPDE further assumes that the nonresolved internal DoF are in thermodynamic equilibrium, mimicking the fundamental hypothesis underlying Onsager’s formulation of Non-Equilibrium Thermodynamics.^29^ Under this hypothesis, we define the particle *bare* entropy ${\mathrm{s̃}}_{i}$ , which is a function of $u_{i}$ and $n_{i}$ at our current level of description. This bare entropy can be related to the density of states $g\left(u_{i},n_{i}\right)$ of the embedded microscopic physical system through

1 $${\mathrm{s̃}}_{i}=k_{B}\;\ln g\left(u_{i},n_{i}\right)$$

In this way, ${\mathrm{s̃}}_{i}$ is a potential for fluctuations,^30^ but cannot be straightforwardly related to the macroscopic thermodynamic entropy of the system. Hence, the foundational principle of the mesoscopic thermodynamic description is that, in equilibrium, the state of a system of *N* interacting mesoparticles is characterized by a probability distribution that, in the canonical ensemble, reads^24^

2 $$P_{eq}\left(\mathrm{\Gamma ̃}\right)\propto e^{-\sum_{i=1}^{N}\left[\frac{p_{i}^{2}}{2m_{i}}+u_{i}-T{\mathrm{s̃}}_{i}\left(u_{i},n_{i}\right)\right]/k_{B}T}$$

where $\mathrm{\Gamma ̃}\equiv \left(\left\{r_{i}\right\},\left\{p_{i}\right\},\left\{u_{i}\right\}\right)$ describes the state of the system, $k_{B}$ is the Boltzmann constant, and *T* is the temperature of the reservoir.

Furthermore, by sheer analogy with macroscopic Thermodynamics,^30^ we can introduce the particle *bare* intensive variables from the relation

3 $$\begin{array}{ll} d{\mathrm{s̃}}_{i} & ={\frac{\partial {\mathrm{s̃}}_{i}}{\partial u_{i}}|}_{n_{i}}du_{i}+{\frac{\partial {\mathrm{s̃}}_{i}}{\partial n_{i}}|}_{u_{i}}du_{i}\\ & \equiv \frac{1}{{\mathrm{\theta ̃}}_{i}}du_{i}-\frac{1}{n_{i}^{2}}\frac{{\mathrm{\pi ̃}}_{i}}{{\mathrm{\theta ̃}}_{i}}dn_{i} \end{array}$$

where

4 $${\frac{1}{{\mathrm{\theta ̃}}_{i}}\equiv \frac{\partial {\mathrm{s̃}}_{i}}{\partial u_{i}}|}_{n_{i}}$$

is a definition of the bare particle temperature ${\mathrm{\theta ̃}}_{i}>0$ , and

5 $$\frac{{\mathrm{\pi ̃}}_{i}}{{\mathrm{\theta ̃}}_{i}}\equiv -n_{i}^{2}{\frac{\partial {\mathrm{s̃}}_{i}}{\partial n_{i}}|}_{u_{i}}$$

is a definition of the bare particle pressure ${\mathrm{\pi ̃}}_{i}$ . Notice that the bare particle temperature is an *estimator* of the reservoir temperature in the canonical ensemble, since its equilibrium average yields

6 $$\left\langle \frac{1}{{\mathrm{\theta ̃}}_{i}}\right\rangle =\frac{1}{T}$$

The bare pressure, on the other hand, is an estimator of the *excess* contribution to the system pressure *P*. For weakly interacting systems, one gets approximately

7 $$\left\langle \frac{{\mathrm{\pi ̃}}_{i}}{{\mathrm{\theta ̃}}_{i}}\right\rangle ≃\frac{P^{ex}}{T}$$

where *P* ^ex^ is the excess pressure defined from the relation *P* = *k*_B_ *T*ρ + *P* ^ex^, with ρ the bulk number density of the system (see Appendix B). The system pressure in DPD fluid is thus obtained as the sum of an excess (internal) contribution, related to the particle pressure, and a kinetic (ideal-gas) contribution, related to the thermal agitation of the mesoparticles. In the limit of large levels of CG, as described by a large number of physical particles in one mesoparticle, ϕ ≫ 1, the latter is negligible and the excess pressure is approximately equal to the system pressure.^17,24^ Notice that, while eq 6 is exact since the equilibrium average does not depend on the particle positions, eq 7 depends on particle positions through the particle local density and is influenced by the local particle arrangements, as discussed in more detail in Appendix B. A comprehensive analysis of the thermodynamic behavior of the GenDPDE model will be addressed elsewhere.

It is important to observe that, since the equilibrium probability distribution is the fundamental object describing the physics of a thermodynamic system at the mesoscale, the change from an *entropic representation* (as in eqs 3–5) to an *energetic representation* does not follow the standard thermodynamic rules that would allow us to simply invert the function s̃=s̃(u,n) to obtain u=u(s̃,n) . The requirement that a change of variable should leave $P_{eq}$ invariant, namely, demanding that $P_{eq}\left(\mathrm{\Gamma ̃}\right)d\mathrm{\Gamma ̃}=P_{eq}\left(\Gamma \right)d\Gamma$ , with Γ≡({**r**_*i*_}, {**p**_*i*_}, {*s*_*i*_}), implies a redefinition of the particle entropy. The probability distribution in the energetic representation reads

8 $$P_{eq}\left(\Gamma \right)\propto e^{-\sum_{i=1}^{N}\left[\frac{p_{i}^{2}}{2m_{i}}+u_{i}\left(s_{i},n_{i}\right)-Ts_{i}\right]/k_{B}T}$$

where *s*_*i*_ is the *dressed* particle entropy, related to the bare entropy through

9 $$s_{i}={\mathrm{s̃}}_{i}-k_{B}\;\ln J$$

with the Jacobian defined from the relation

10 $$J\left(u_{i},n_{i}\right)={\frac{\partial s_{i}}{\partial u_{i}}|}_{n_{i}}>0$$

Therefore, it is important to realize that, unlike in the macroscopic world, mesoscopic thermodynamic relations transform as distributions. The relation eq 3 thus becomes

11 $$\begin{array}{ll} du_{i} & ={\frac{\partial u_{i}}{\partial s_{i}}|}_{n_{i}}ds_{i}+{\frac{\partial u_{i}}{\partial n_{i}}|}_{s_{i}}dn_{i}\\ & \equiv \theta_{i}ds_{i}+\frac{\pi_{i}}{n_{i}^{2}}dn_{i} \end{array}$$

where

12 $$\theta_{i}\equiv {\frac{\partial u_{i}}{\partial s_{i}}|}_{n_{i}}$$

is a definition of the dressed particle temperature θ_*i*_ > 0, and

13 $$\pi_{i}\equiv n_{i}^{2}{\frac{\partial u_{i}}{\partial n_{i}}|}_{s_{i}}$$

is a definition of the dressed particle pressure π_*i*_. The dressed temperature and pressure are also estimators of the system temperature and excess pressure, yielding (see Appendix B)

14 $$\left\langle \theta_{i}\right\rangle =T$$

15 $$\left\langle \pi_{i}\right\rangle ≃P^{ex}$$

Thus, eqs 6, 7, 14 and 15 relate mesoscopic thermodynamic quantities to their macroscopic counterparts.

Finally, for the sake of completeness, using eq 9, a relationship between bare and dressed intensive variables can be found, namely

16 $$\frac{1}{{\mathrm{\theta ̃}}_{i}}=\frac{1}{\theta_{i}}+k_{B}{\frac{\partial }{\partial u_{i}}\ln\frac{1}{\theta_{i}}|}_{n_{i}}$$

for the temperature, and

17 $$\frac{{\mathrm{\pi ̃}}_{i}}{{\mathrm{\theta ̃}}_{i}}=\frac{\pi_{i}}{\theta_{i}}-k_{B}n_{i}^{2}{\frac{\partial }{\partial n_{i}}\ln\frac{1}{\theta_{i}}|}_{u_{i}}$$

for the pressure. As the last term in the right-hand side of eqs 16 and 17 is inversely proportional to the particle heat capacity *C*_V_, the difference between bare and dressed variables is of the order of magnitude of the fluctuations, *k*_B_/*C*_V_, and vanishes as the size of the mesoparticle increases.

Both bare and dressed variables are needed in the formulation of the GenDPDE algorithm in Section 3.

### 2.2 Local Density

Traditionally, in DPD-like approaches, the local density has been estimated from the expression^16,17,21^

18 $$n_{i}^{b}\equiv \sum_{j\neq i}w\left(r_{ij}\right)$$

Here, the superscript *b* denotes *bare*, to distinguish the classical calculation *n* ^*b*^ from that introduced in this article, which we denote as *n*. In eq 18, *w*(*r*_*ij*_) is a smooth, non-negative, monotonically decreasing, spherically symmetric weighting function, vanishing for an interparticle distance, *r*_*ij*_ ≡|**r**_*ij*_| = |**r**_*i*_ – **r**_*j*_|, such that *r*_*ij*_ ≥ *R*_cut_, where *R*_cut_ is the cutoff radius for this property. This weighting function is furthermore normalized so that

19 ∫drw(r)=1

From the definition eq 18, it is clear that *n*_*i*_^*b*^({**r**_*ij*_}) is a function of the positions of all the mesoparticles *j* that can be found inside the sphere of radius *R*_cut_ and centered at **r**_*i*_ at a given instant of time. Hence, *n*_*i*_^*b*^ depends on the neighboring environment and provides an estimation of the particle volume from $V_{i}=1/n_{i}^{b}$ . However, it is important to notice that, since *w*(*r*_*ij*_) is an inhomogeneous function, the calculated value of *n*_*i*_^*b*^ is mainly determined by the nearest neighbors, whereas the contribution of more distant mesoparticles becomes gradually smaller. Moreover, the use of eq 18 for the volume estimate causes the loss of volume additivity, since $V\neq \sum_{i}V_{i}$ , *V* being the system volume. The magnitude of difference between $\sum_{i}V_{i}$ and the nominal value *V* is more or less significant depending on the local arrangement of particles, on the functional form of the kernel *w*(*r*_*ij*_), and on the selected cutoff distance. To clarify this latter statement, let us consider two limiting cases, namely, an ideal gas and a crystalline structure. In an ideal gas, particles are free to diffuse and distribute over the volume without positional correlations. Thus, the estimated local density is, on average, the same at every point in space and does not vary with *R*_cut_. In crystals, on the other hand, particles occupy well-defined regular positions in space. Due to the inhomogeneity of *w*(*r*_*ij*_) and according to the chosen value of *R*_cut_, we may thus obtain significantly different results depending on the number and relative positions of neighboring particles considered in the evaluation of the local density of the crystal. A similar problem occurs in fluids with interacting particles, far from the ideal gas limit, which also develop a local structure, albeit less rigid than in solids. Thus, the use of eq 18 negatively affects the density calculation when structured systems are considered and *R*_cut_ is slightly larger than the size of the correlation range between particles. The consequences of such inaccuracies are analyzed in more detail in Section 4.

#### 2.2.1 New Perspective for the Estimation of the Particle Volume

To avoid such conundrums, we revise the determination of the particle volume, starting from a definition that satisfies the additivity requirement. Additivity can be ensured by use of, e.g., a three-dimensional Voronoi tessellation,^31^ which determines the particle volume from a geometrical division of space in volumes containing one particle. Another possible route is to follow the *partition-of-unity* approach discussed by Flekkøy et al.^32^ In both cases, however, the volume determination is computationally expensive and induces severe complications in the definition of the interparticle forces, as the volume additivity represents a global constraint. We shall consider the partition-of-unity as the starting point to generate a good approximation for the particle volume, although we later sacrifice global volume additivity, but allow a fast and simple evaluation of this quantity. Crucially, we will prove that the proposed procedure corrects the problem of the spurious local rearrangement of particles.

Let us focus again on the weighting function introduced in eq 18. More precisely, we consider the quadratic weighting function

20 $$w\left(r\right)=\frac{15}{2\pi R_{cut}^{3}}{\left(1-\frac{r}{R_{cut}}\right)}^{2}$$

as it is a conventional choice in DPD-like methods.^16,17,21^ Next, let us define the set of functions φ_*i*_, based on *w*(*r*), which introduce a partition of the unity, i.e.

21 $$\varphi_{i}\left(r\right)\equiv \frac{w\left(|r-r_{i}|\right)}{\sum_{j}w\left(|r-r_{j}|\right)}$$

In eq 21, the summation in the denominator runs over all mesoparticles, including *j* = *i*. By construction, we thus have that

22 $$\sum_{i}\varphi_{i}\left(r\right)=1$$

Consequently, the set of functions φ_*i*_ can be exploited to construct a partition of any field defined in the volume *V*. In particular, we can express the volume itself as

23 $$V=\int dr1=\int dr\sum_{i}\varphi_{i}\left(r\right)\equiv \sum_{i}V_{i}$$

where we have defined the particle volume

24 $$V_{i}\equiv \int dr\varphi_{i}\left(r\right)$$

Equation 21 can also be reformulated by explicitly separating the *i*th-particle contribution in the denominator, namely

25 $$\varphi_{i}\left(r\right)=\frac{w\left(|r-r_{i}|\right)}{w\left(|r-r_{i}|\right)+\sum_{j\neq i}w\left(|r-r_{j}|\right)}$$

However, building an analytical solution for eq 24 is impaired by the second term in the denominator of eq 25, which contains the relative coordinates between neighboring particles. To better understand the nature of this second term, and the approximations that we will introduce, let us analyze it from a mean-field perspective. To this end, we first define the instantaneous *primitive* local density field ${\mathrm{n̂}}_{i}^{b}\left(r\right)$ , namely

26 $$\begin{array}{ll} {\mathrm{n̂}}_{i}^{b}\left(r\right) & \equiv \sum_{j\neq i}w\left(|r-r_{j}|\right)\\ & =\int dr^{\prime }w\left(|r-r^{\prime }|\right)\sum_{j\neq i}\delta \left(r^{\prime }-r_{j}\right)\\ & \equiv \int dr^{\prime }w\left(|r-r^{\prime }|\right){\mathrm{\rho ̂}}_{i}\left(r^{\prime }\right) \end{array}$$

based on the traditional DPD estimation of the local particle density. Here, the instantaneous number density, ${\mathrm{\rho ̂}}_{i}\left(r^{\prime }\right)\equiv \sum_{j\neq i}\delta \left(r^{\prime }-r_{j}\right)$ is defined, where the subscript indicates that this density is actually *conditioned* by the exclusion of the central particle *i*. The mean field approximation for this field follows from a relation that takes into account such a correlation, i.e.

27 $$\begin{array}{ll} \left\langle \mathrm{\rho ̂}\left(r\right){\mathrm{n̂}}_{i}^{b}\left(r\right)\right\rangle & =\int dr^{\prime }w\left(|r-r^{\prime }|\right)\times \\ & \times \left\langle \sum_{i,j\neq i}\delta \left(r-r_{i}\right)\delta \left(r^{\prime }-r_{j}\right)\right\rangle \\ & =\int dr^{\prime }w\left(|r-r^{\prime }|\right)\rho \left(r\right)\rho \left(r^{\prime }\right)g\left(r-r^{\prime }\right)\\ & \equiv \rho \left(r\right)n\left(r\right) \end{array}$$

where $\rho \left(r\right)\equiv \left\langle \mathrm{\rho ̂}\left(r\right)\right\rangle =\left\langle \sum_{i=1}^{N}\delta \left(r-r_{i}\right)\right\rangle$ represents the average mesoparticle density and *g*(**r** – **r**′) stands for the pair distribution function for a translationally invariant system, defined from^11^

28 $$\rho \left(r\right)\rho \left(r^{\prime }\right)g\left(r-r^{\prime }\right)\equiv \left\langle \sum_{i,j\neq i}\delta \left(r-r_{i}\right)\delta \left(r^{\prime }-r_{j}\right)\right\rangle$$

Here, ⟨··· ⟩ stands for the equilibrium average. Remarkably, eq 27 reveals that, even in the mean-field approximation, the density *n*(**r**) depends on the local structure of the system through *g*(**r** – **r**′). Therefore, introducing Δ**r** ≡ **r** – **r**_*i*_ to place the center of coordinates on **r**_*i*_, and using the mean-field approximation in a translationally invariant and isotropic system, we can write

29 $$\sum_{j\neq i}w\left(|\Delta r+r_{i}-r_{j}|\right)≃\int dr^{\prime }w\left(|\Delta r-r^{\prime }|\right)\rho g\left(|r^{\prime }|\right)$$

Notice that, in a locally structured system, particles create *correlation holes* in their vicinities, due to the repulsive nature of the interaction potentials. We denote the characteristic range of the correlation hole by *l*. In a homogeneous system with density-dependent potentials, the size of the correlation hole is determined by the overall density, namely, *l* ∼ ρ^–1/3^. Therefore, the condition *l* < *R*_cut_ is required for a meaningful evaluation of the primitive local density from eq 26. The existence of the correlation hole is the main reason behind the poor volume estimation yielded by eq 18 when *R*_cut_ is only slightly larger than *l*.

With the aim of finding an analytical expression for $V_{i}$ from eq 24, let us now introduce suitable approximations to eq 25 by considering several relevant cases. We start by analyzing the situation in which *R*_cut_ ≫ *l*. In this case, the weight of the correlation hole in the integration of eq 29 is subdominant, and we can write

30 $$\begin{array}{ll} \int dr^{\prime }w\left(|\Delta r-r^{\prime }|\right)\rho g\left(r^{\prime }\right) & =\rho +\int dr^{\prime }w\left(|\Delta r-r^{\prime }|\right)\rho h\left(r^{\prime }\right)≃\\ & ≃\rho \left(1+w\left(|\Delta r|\right)\int dr^{\prime }h\left(r^{\prime }\right)\right)≃\\ & ≃\rho \left(1-w\left(|\Delta r|\right)\frac{1}{\rho }\right)) \end{array}$$

where *h*(**r**) = *g*(**r**) – 1 is the total correlation function^11^ and ∫d**r** *h*(**r**) = – 1/ρ, when *R*_cut_/*l* → ∞. Thus, using this result into eq 24, we find that

31 $$V_{i}≃\int d\Delta r\frac{w\left(|\Delta r|\right)}{\rho }=\frac{1}{\rho }$$

Second, we analyze the more stringent case of *R*_cut_ ∼ *l*, which is also the case of major interest. Let us consider |Δ**r**|≪ *l* < *R*_cut_ in the first place. From eq 25, one can write

32 $$\begin{array}{ll} \varphi_{i}\left(\Delta r\right) & =\frac{w\left(|\Delta r|\right)}{w\left(|\Delta r|\right)+\sum_{j\neq i}w\left(|\Delta r+r_{i}-r_{j}|\right)}≃\\ & ≃\frac{w\left(|\Delta r|\right)}{w\left(|\Delta r|\right)+\sum_{j\neq i}w\left(|r_{i}-r_{j}|\right)}=\\ & =\frac{w\left(|\Delta r|\right)}{w\left(|\Delta r|\right)+n_{i}^{b}} \end{array}$$

where

33 $$\begin{array}{ll} n_{i}^{b}\left(r\right) & =\int dr^{\prime }w\left(|r-r^{\prime }|\right)\rho g\left(|r-r^{\prime }|\right)=\\ & =\rho \left[1+\int dr^{\prime }w\left(|r-r^{\prime }|\right)h\left(|r-r^{\prime }|\right)\right]∼\\ & ∼\rho \delta^{3} \end{array}$$

with δ ≡ 1 – *l*/*R*_cut_ ≪ 1. The contribution of this region to the volume calculation can thus be written as

34 $$\begin{array}{ll} V_{i}^{<} & {≃\int }_{<}d\Delta r\frac{w\left(|\Delta r|\right)}{w\left(|\Delta r|\right)+\rho \delta^{3}}=\\ & =4\pi R_{cut}^{3}\int_{0}^{l/R_{cut}}dxx^{2}\frac{{\left(1-x\right)}^{2}}{{\left(1-x\right)}^{2}+\frac{2\pi R_{cut}^{3}}{15}\rho \delta^{3}}=\\ & =\frac{4\pi }{3}l^{3} \end{array}$$

Effectively, the second term in the denominator can be neglected as the dominant term is (1 – *x*)^2^ → δ^2^ at the limit of the integration interval. In addition, notice that the product *R*_cut_^3^ρ ∼ *R*_cut_^3^/*l* ^3^ ∼ 1/(1 – δ)^3^ ∼ 1 + 3δ. Including the next-order corrections to this leading behavior, one obtains

35 $$V_{i}^{<}≃\frac{4\pi }{3}l^{3}+O\left(R_{cut}^{3}\delta^{3/2}\right)$$

Notice that the dominant contribution assigns the volume of the particle to a volume proportional to that of the correlation hole, as it should be expected, with additional subdominant terms. The difference between *l* and *R*_cut_ is of order δ.

Instead, when *l* < |Δ**r**| < *R*_cut_, the integration domain over the particle positions has the form of the blue circle as sketched in Figure 1.Then, the mean field approximation of the integral yields

36 $$\begin{array}{ll} \int dr^{\prime } & \rho g\left(r^{\prime }\right)w\left(|\Delta r-r^{\prime }|\right)=\\ & =\rho \left(1+\int dr^{\prime }h\left(r^{\prime }\right)w\left(|\Delta r-r^{\prime }|\right)\right)∼\\ & ∼\rho -\rho \delta^{3} \end{array}$$

**Figure 1:**
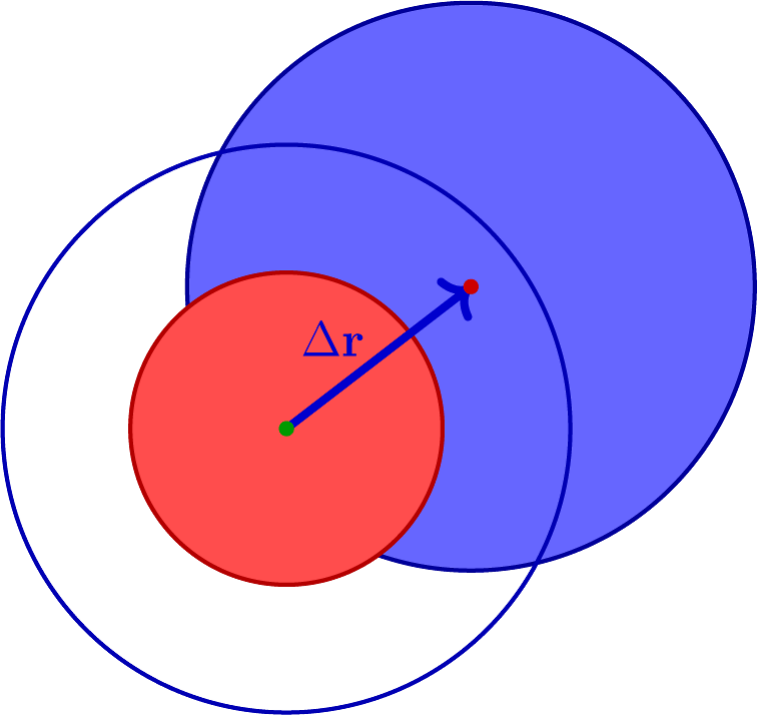
Integration domain for the calculation of the particle volume. Δ**r** is a field point near the weighting function cutoff range, the red circle represents the correlation hole and the blue area is the region within the integration domain where *g*(*r*) ≃ 1.

Although this part is apparently dominant for δ ≪ 1, its contribution to the volume is limited by the fact that *w*(Δ**r**) ∼ δ^2^ in this region. Hence

37 $$\begin{array}{ll} \varphi_{i}\left(\Delta r\right) & ≃\frac{w\left(|\Delta r|\right)}{w\left(|\Delta r|\right)+\rho -\rho \delta^{3}}≃\\ & ≃\frac{w\left(|\Delta r|\right)}{\rho } \end{array}$$

and the contribution to the particle volume reads

38 $$\begin{array}{ll} V_{i}^{>}≃ & 4\pi R_{cut}^{3}\int_{l/R_{cut}}^{1}x^{2}\frac{{\left(1-x\right)}^{2}}{\frac{2\pi R_{cut}^{3}\rho }{15}}∼\\ ∼ & \frac{10}{\rho }\left[1-{\left(1-\delta \right)}^{3}\right]∼\frac{30}{\rho }\delta \end{array}$$

Thus, the dominant contribution to the calculation of the volume in the limit *R*_cut_ ∼ *l* is obtained by adding the $V_{i}^{<}$ and $V_{i}^{>}$ contributions, and reads

39 $$V_{i}≃\frac{4\pi }{3}l^{3}-O\left[R_{cut}^{3}\left(\delta^{3/2}-\frac{1}{\rho R_{cut}^{3}}\delta \right)\right]$$

In view of this analysis, we propose the use of the approximate function

40 $$\varphi_{i}^{*}\left(\Delta r\right)≃\frac{w\left(|\Delta r|\right)}{w\left(|\Delta r|\right)+n_{i}^{b}}$$

to determine the particle volume. On the one hand, the approximate φ_i_^*^ provides an exact estimate of the particle volume for large values of *R*_cut_ ≫ *l*. Effectively, in this limit, using a mean field perspective as in eq 30, one finds

41 $$n_{i}^{b}≃\rho \left(1-w\left(0\right)\frac{1}{\rho }\right)∼\rho \left(1-\frac{1}{\rho R_{cut}^{3}}\right)$$

where the second term in the parentheses is negligibly small as *ρR*_cut_^3^≫1. Hence, the integration of φ_i_^*^ in this limit produces the expected result, $V_{i}=1/\rho$ . On the other hand, in the limit *R*_cut_ ∼ *l*, φ_i_^*^ provides a reasonable estimate of the order of the actual particle volume, as we have seen that the leading contribution is of the order of *l* ^3^, although corrections need to be considered.

The real advantage in the use of φ_i_^*^ is that an analytical approximation of the integral eq 24 can be obtained, which allows to accurately estimate $V_{i}$ without significantly increasing the computational effort, as would be the case with tessellation approaches. In particular, with the quadratic kernel eq 20, an explicit solution of eq 24 using eq 40 yields

42 $$\begin{array}{ll} V_{i}= & \frac{4\pi }{3}{\mathrm{R̃}}_{cut}^{3}-4\pi {\mathrm{R̃}}_{cut}^{3}\sqrt{k_{i}}\{\left(1-k_{i}\right)\arctan\left(\frac{1}{\sqrt{k_{i}}}\right)\\ & +\sqrt{k_{i}}\left[1-\ln\left(\frac{k_{i}+1}{k_{i}}\right)\right]\} \end{array}$$

with

43 $$k_{i}=\frac{2\pi }{15}{\mathrm{R̃}}_{cut}^{3}n_{i}^{b}$$

In eqs 42 and 43, ${\mathrm{R̃}}_{cut}\equiv R_{cut}/f_{cut}$ , where *f*_cut_ > 1 is a scaling factor for the original cutoff radius, introduced with the aim of accounting for the aforementioned corrections to the volume calculation. It is important to realize that the rescaling of *R*_cut_ only affects the evaluation of *k*_*i*_ as well as the prefactor of the volume expression in eqs 42 and 43. The direct measure of *n*_*i*_^*b*^ is still of the range *R*_cut_ and is not varied. Therefore, the computational effort is independent of the correction introduced in the evaluation of the volume using ${\mathrm{R̃}}_{cut}$ from *n*_*i*_^*b*^.

#### 2.2.2 Analysis of the Case of a Two-Dimensional Hexagonal Lattice

To test the quality of the approximations introduced in the determination of the particle volume, let us consider a hexagonal lattice with vectors **a** = *l* (1, 0) and $b=l\left(1/2,\sqrt{3}/2\right)$ , where *l* is the interparticle distance in arbitrary units. Hence, any particle in the lattice is located at a vector **r**_αβ_ = α**a** + β**b**, with α and β arbitrary integers. For $l=\sqrt{2/\sqrt{3}}≃1.0746$ the particle density is exactly ρ = 1. The density of the lattice can thus be changed by varying *l*. For the two-dimensional case, the volume function equivalent to eq 42 is written as

44 $$\begin{array}{ll} V_{i} & =\pi {\mathrm{R̃}}_{cut}^{2}-2\pi {\mathrm{R̃}}_{cut}^{2}\sqrt{k}[\arctan\left(\frac{1}{\sqrt{k}}\right)\\ & -\sqrt{k}\frac{1}{2}\ln\left(\frac{1}{k}+1\right)] \end{array}$$

with

45 $$k_{i}=\frac{\pi }{6}{\mathrm{R̃}}_{cut}^{2}n_{i}^{b}$$

and $w\left(r\right)=6{\left(1-r/R_{cut}\right)}^{2}/\pi$ .

Thus, let us consider a fixed value of *R*_cut_ = 2.1564, and calculate the estimation of the particle volume by homothetically changing the interparticle distance without disturbing the hexagonal arrangement. In Figure 2 we show the comparison between the exact particle volume, the traditional estimation through *n*_*i*_^*b*^ (cf. eq 18), namely $V_{i}^{b}=1/n_{i}^{b}$ , and the new estimate from eq 44 for different values of *f*_cut_. Notice that with no scaling (*f*_cut_ = 1), the new estimate of $V_{i}$ slightly improves the traditional one, but the best result is found at *f*_cut_ = 1.8, for which the curve exactly matches the geometrical evaluation of the volume. The profiles are rather sensitive to the scaling factor, which has to be carefully chosen for each system to be analyzed, but it is remarkable that the matching can be achieved by the use of one single adjustable parameter, whereas in the same region, the traditional method overestimates the particle volume by 100%. Therefore, for locally structured systems, the evaluation of *n*_*i*_^*b*^ from the actual configuration around a central particle *i* can serve to obtain an accurate estimate of the volume it occupies, if the correlation hole is properly accounted for. This is particularly relevant for the determination of the particle internal state, since it depends on the local density estimate and, consequently, on the accuracy and performance of the model.

**Figure 2:**
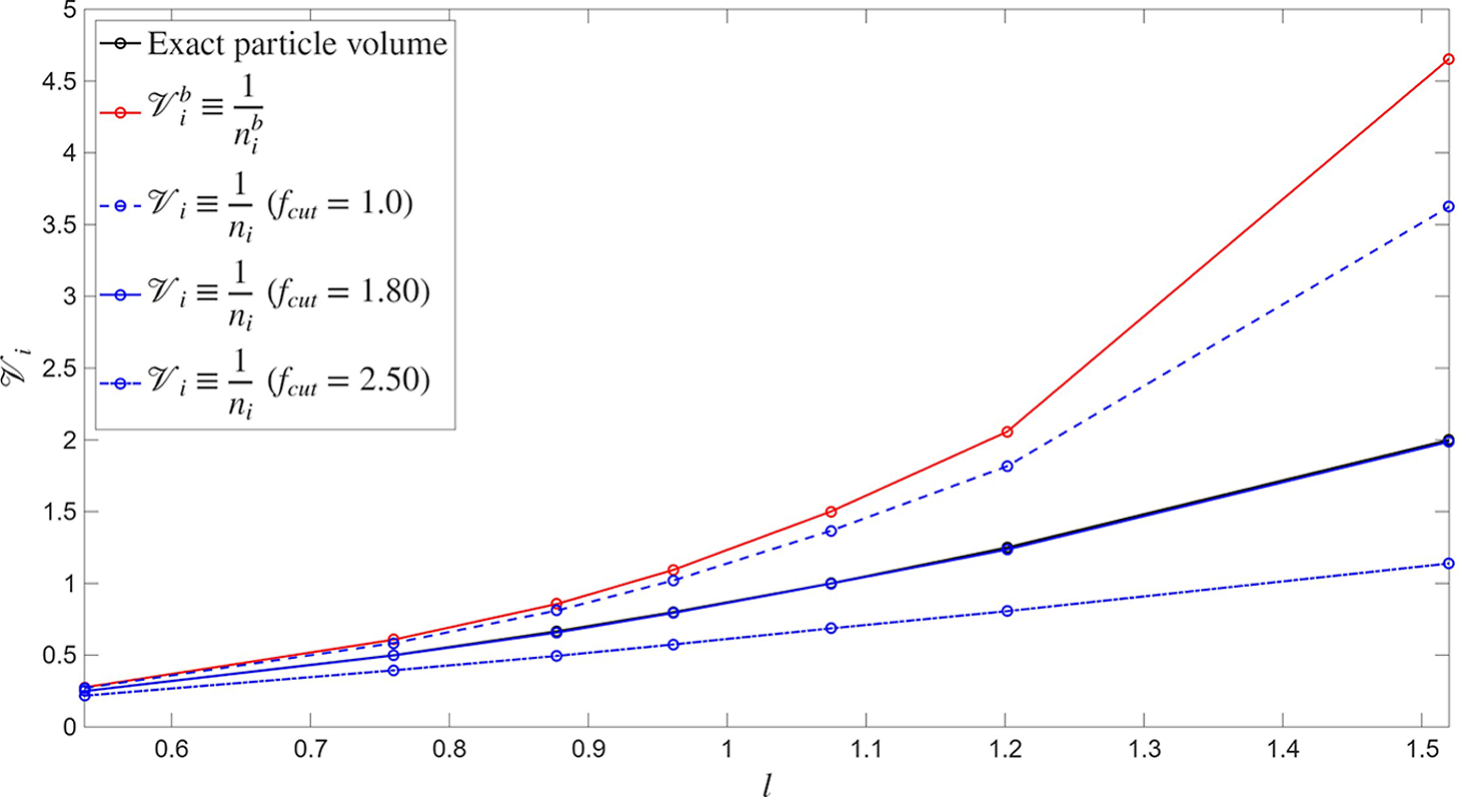
Comparison of the different measures of the particle volume (surface) in a two-dimensional hexagonal array for different values of the characteristic lattice spacing *l*, with *R*_cut_ = 2.1564. We compare the prediction using the traditional *n*_*i*_^*b*^ estimator with eq 44 for *f*_cut_ = 1, 1.80, and 2.50 and the geometrically calculated volume. Notice that with *f*_cut_ = 1.80 eq 44 yields an almost exact evaluation of the particle volume over the whole range.

## 3 The GenDPDE Algorithm

In this section, we briefly introduce the GenDPDE algorithm. The reader is again referred to refs 24 and 25 for a comprehensive derivation. We start by presenting the complete set of EoM that describe the dynamics of the system. Then, we introduce the EoS defining the local particle thermodynamics. The last part of this section is devoted to the computational details of our numerical simulations.

### 3.1 System Dynamics: GenDPDE Equations of Motion

The discrete EoM describing the dynamics of a GenDPDE system stem from the original DPDE algorithm,^22^ with the addition of an equation describing the time evolution of the mesoparticle internal energy, which results from total energy balance.^24^ They read

46 $$r_{i}^{\prime }=r_{i}+\frac{p_{i}}{m_{i}}\delta t$$

47 $$p_{i}^{\prime }=p_{i}+\sum_{j\neq i}f_{ij}^{C}\delta t+\sum_{j\neq i}f_{ij}^{D}\delta t+\sum_{j\neq i}\delta p_{ij}^{R}$$

48 $$\begin{array}{ll} u_{i}^{\prime } & =u_{i}-\frac{1}{2}\sum_{j\neq i}\left(\frac{p_{i}}{m_{i}}-\frac{p_{j}}{m_{j}}\right)\cdot f_{ij}^{C}\delta t\\ & -\frac{1}{2}\sum_{j\neq i}\left(\frac{p_{i}}{m_{i}}-\frac{p_{j}}{m_{j}}\right)\cdot f_{ij}^{D}\delta t\\ & -\frac{1}{2}\sum_{j\neq i}\left(\frac{p_{i}}{m_{i}}-\frac{p_{j}}{m_{j}}\right)\cdot \delta p_{ij}^{R}\\ & -\frac{1}{2m_{i}}\sum_{j\neq i}\sum_{l\neq i}\delta p_{ij}^{R}\cdot \delta p_{il}^{R}\\ & +\sum_{j\neq i}{\mathrm{q̇}}_{ij}\delta t+\sum_{j\neq i}\delta u_{ij}^{R} \end{array}$$

Here, the nonprimed variables refer to a time instant *t*, while primed variables refer to the instant *t* + δ*t*, δ*t* being the time step. The use of a discrete time-step in the algorithm precludes any ambiguity in the interpretation of the stochastic EoM, which are written in explicit (causal) form. In eq 47, the term **f**_*ij*_^*C*^ represents the conservative force acting between a pair of particles, *i* and *j*, and is defined as

49 $$\begin{array}{ll} f_{ij}^{C} & =-{\frac{\partial u_{i}}{\partial n_{i}}|}_{s_{i}}{\frac{\partial n_{i}}{\partial r_{j}}+\frac{\partial u_{j}}{\partial n_{j}}|}_{s_{j}}\frac{\partial n_{j}}{\partial r_{i}}\\ & =-\frac{\pi_{i}}{n_{i}^{2}}\frac{\partial n_{i}}{\partial r_{j}}+\frac{\pi_{j}}{n_{j}^{2}}\frac{\partial n_{j}}{\partial r_{i}} \end{array}$$

The last line of eq 49 has been obtained using the definition eq 13. The explicit expression for the partial derivative of the density with respect to the position vector depends on the chosen definition of the local density. If eq 18 is considered, eq 49 simply reads^24^

50 $$f_{ij}^{C}=-\left(\frac{\pi_{i}}{n_{i}^{2}}+\frac{\pi_{j}}{n_{j}^{2}}\right)\frac{dw_{ij}}{dr_{ij}}e_{ij}$$

with **e**_*ij*_ = **r**_*ij*_/*r*_*ij*_ the separation-distance unit vector, and *w*_*ij*_ ≡ *w*(*r*_*ij*_). If, on the other hand, eq 42 is used to define the local density as $n_{i}=1/V_{i}$ , an additional contribution appears in the derivative. Indeed, since *n*_*i*_ now depends on **r**_*j*_ through *n*_*i*_^*b*^, we can write

51 $$\frac{\partial n_{i}}{\partial r_{j}}=\zeta_{i}\frac{\partial n_{i}^{b}}{\partial r_{j}}$$

where

52 $$\zeta_{i}\equiv \frac{\partial n_{i}}{\partial n_{i}^{b}}=\frac{\partial \left(1/V_{i}\right)}{\partial n_{i}^{b}}=-\frac{1}{V_{i}^{2}}\frac{\partial V_{i}}{\partial k_{i}}\frac{\partial k_{i}}{\partial n_{i}^{b}}$$

with

53 $$\begin{array}{ll} \frac{\partial V_{i}}{\partial k_{i}} & =-4\pi {\mathrm{R̃}}_{cut}^{3}[\frac{3}{2}+\frac{1-3k_{i}}{2\sqrt{k_{i}}}\arctan\left(\frac{1}{\sqrt{k_{i}}}\right)\\ & -\ln\left(\frac{k_{i}+1}{k_{i}}\right)] \end{array}$$

and

54 $$\frac{\partial k_{i}}{\partial n_{i}^{b}}=\frac{2\pi }{15}{\mathrm{R̃}}_{cut}^{3}$$

in three dimensions. An analogous derivation can be carried out for the term ∂*n*_*j*_/∂**r**_*i*_. Thus, in this case, eq 49 becomes

55 $$f_{ij}^{C}=-\left(\frac{\pi_{i}}{n_{i}^{2}}\zeta_{i}+\frac{\pi_{j}}{n_{j}^{2}}\zeta_{j}\right)\frac{dw_{ij}}{dr_{ij}}e_{ij}$$

Notice that, when *n* = *n* ^*b*^, ζ = 1 and one recovers eq 50. A crucial point that emerges from the derivation of eq 55 is that the new local density definition does not bring along any severe complications in the evaluation of the interparticle conservative forces, which remain pairwise additive. As proven in Sec. [Sec sec4], this also implies that eq 42 can be implemented numerically without any significant additional computational cost. The contribution **f**_*ij*_^*D*^ in eq 47 represents the usual DPD friction force, i.e.

56 $$f_{ij}^{D}=-\gamma_{ij}\left(\frac{p_{i}}{m_{i}}-\frac{p_{j}}{m_{j}}\right)\cdot e_{ij}e_{ij}$$

where γ_*ij*_ = γ ω^*p*^(*r*_*ij*_), γ being the mesoscopic friction coefficient and ω^*p*^(*r*_*ij*_) being a weighting function with the same properties as *w*(*r*_*ij*_) and vanishing for *r*_*ij*_ ≥ *R*_cut_^*p*^, with *R*_cut_^*p*^ the corresponding cutoff radius. Unlike *w*(*r*_*ij*_), however, ω^*p*^(*r*_*ij*_) may not be normalized. Finally, the term δ**p**_*ij*_^*R*^ in eq 47 represents the random contribution to the particle momentum, related to the dissipative contribution through the suitable FD theorem^24^ and reading

57 $$\delta p_{ij}^{R}=\sqrt{k_{B}\left(\theta_{i}+\theta_{j}\right)\gamma_{ij}}\Omega_{ij}^{p}e_{ij}\delta t^{1/2}$$

with Ω_*ij*_^*p*^ a normalized Gaussian number satisfying

58 $$\left\langle \Omega_{ij}^{p}\right\rangle =0$$

59 $$\left\langle \Omega_{ij}^{p}\left(t\right)\Omega_{kl}^{p}\left(t^{\prime }\right)\right\rangle =\left(\delta_{ik}\delta_{jl}-\delta_{il}\delta_{jk}\right)\delta_{tt^{\prime }}$$

In these expressions, δ_*ij*_ is the Kronecker symbol, equal to 1 if *i* = *j* and 0, otherwise. In turn, δ_*tt*′_ is 1 if *t* and *t*′ belong to the same time interval. In eq 48, the term ${\mathrm{q̇}}_{ij}$ is the heat flux between mesoparticles *i* and *j*, defined as

60 $${\mathrm{q̇}}_{ij}=-\kappa_{ij}\left(\frac{1}{{\mathrm{\theta ̃}}_{j}}-\frac{1}{{\mathrm{\theta ̃}}_{i}}\right)$$

where κ_*ij*_ = κ ω^*u*^(*r*_*ij*_), κ being the mesoscopic thermal conductivity and ω^*u*^(*r*_*ij*_) is a weighting function with the same properties as ω^*p*^(*r*_*ij*_), and vanishing for *r*_*ij*_ ≥ *R*_cut_^*u*^, with *R*_cut_^*u*^ the respective cutoff range. Lastly, the term *δu*_*ij*_^*R*^ in eq 48 is the random contribution to the internal energy, related to the interparticle heat flux through the appropriate FD theorem^24^ which reads

61 $$\delta u_{ij}^{R}=\sqrt{2k_{B}\kappa_{ij}}\Omega_{ij}^{u}e_{ij}\delta t^{1/2}$$

with Ω_*ij*_^*u*^ a normalized Gaussian number also satisfying the properties in eqs 58 and 59.

### 3.2 Internal Thermodynamic Model: Mesoscopic Equations of State

The EoM eqs 46–48 must be supplemented by EoS that describe the internal thermodynamics of the mesoparticle and provide explicit relationships between intensive and extensive particle variables, serving as closure laws for the set of equations. In this work, the modified Tait EoS for weakly compressible fluids^28^ has been chosen as the representative model for the mesoparticle thermodynamics. This EoS is suitable for weakly compressible fluids and is widely used in other Lagrangian-based approaches, such as Smoothed Particle Hydrodynamics (SPH).^33,34^ Thus, it is an appropriate choice to describe systems in liquid state, which is one of the main target applications of the ideas developed in the present article. This equation relates the particle pressure to the local density as

62 $$\pi_{i}=\pi_{0}+B_{T}\left[{\left(\frac{n_{i}}{n_{0}}\right)}^{\gamma_{T}}-1\right]$$

where γ_T_ is a fitting parameter that depends on the considered substance as well as on the temperature, π_0_ and *n*_0_ are reference values for the pressure and the number density respectively, and

63 $$B_{T}\equiv \frac{1}{\gamma_{T}\kappa_{T}^{0}}$$

with κ_T_^0^ the isothermal compressibility of the substance at the reference state. Notice that the LTh compressibility is defined from eq 62, according to the relation

64 $$\kappa_{T}\equiv -\frac{1}{V}{\frac{\partial V}{\partial \pi }|}_{\theta }=\frac{1}{n}{\frac{\partial n}{\partial \pi }|}_{\theta }=\kappa_{T}^{0}{\left(\frac{n_{0}}{n}\right)}^{\gamma_{T}}$$

Therefore, at the reference state *n* = *n*_0_ and the compressibility of the LTh model equals that of the physical reference state. Although the validity of Tait’s EoS is limited to small deviations around a reference state used to fit the parameters, it is sufficient to describe well the pressure caused by fluctuating densities due to thermal agitation, as they will be small.

Considering eq 62, the particle Helmholtz free energy *f*_*i*_ can be derived from the relation $\pi_{i}=n_{i}^{2}{\frac{\partial f_{i}}{\partial n_{i}}|}_{\theta_{i}}$ . Upon integration, we thus have

65 $$f_{i}=\frac{B_{T}-\pi_{0}}{n_{i}}+\frac{B_{T}}{\left(\gamma_{T}-1\right)n_{0}}{\left(\frac{n_{i}}{n_{0}}\right)}^{\gamma_{T}-1}+\Phi \left(\theta_{i}\right)$$

where Φ­(θ_*i*_) is a function of the particle temperature only. We propose

66 $$\Phi \left(\theta_{i}\right)\equiv -\frac{1}{2}C_{V,i}\theta_{i}\;\ln\left(1+{\left(\frac{\theta_{i}}{\theta_{0}}\right)}^{2}\right)$$

with θ_0_ a reference value for the temperature and *C*_V,*i*_ a parameter related to the constant-volume heat capacity of the mesoparticle. With the definition eq 66, the particle entropy can be derived from the relation $s_{i}={-\frac{\partial f_{i}}{\partial \theta_{i}}|}_{n_{i}}$ , as

67 $$s_{i}=\frac{1}{2}C_{V,i}\left[\frac{2}{1+{\left(\theta_{0}/\theta_{i}\right)}^{2}}+\ln\left(1+{\left(\frac{\theta_{i}}{\theta_{0}}\right)}^{2}\right)\right]$$

Notice that the chosen expression for Φ­(θ_*i*_) ensures that *s*_*i*_ is bounded as θ_*i*_ → 0, and that $\frac{\partial s_{i}}{\partial \theta_{i}}\to 0$ in the same limit, thus satisfying the Third Law of Thermodynamics. Finally, the particle internal energy results from *u*_*i*_ = *f*_*i*_ + θ_*i*_ *s*_*i*_ and reads

68 $$u_{i}=\Psi \left(n_{i}\right)+C_{V,i}\frac{\theta_{i}}{1+{\left(\theta_{0}/\theta_{i}\right)}^{2}}$$

with

69 $$\Psi \left(n_{i}\right)=\frac{B_{T}-\pi_{0}}{n_{i}}+\frac{B_{T}}{\left(\gamma_{T}-1\right)n_{0}}{\left(\frac{n_{i}}{n_{0}}\right)}^{\gamma_{T}-1}$$

As the particle free energy shown in eq 65 is split into two terms, Ψ­(*n*_*i*_) and Φ­(θ_*i*_), the former actually becomes a potential for the pressure as $\pi_{i}=n_{i}^{2}\frac{d\Psi_{i}}{dn_{i}}$ , which in this case is independent of the temperature, although this dependence could be present in other models.^24^ An important property of Ψ­(*n*) is that it has a minimum at a density

70 $$n_{\min}=n_{0}{\left(1-\frac{\pi_{0}}{B_{T}}\right)}^{1/\gamma_{T}}$$

At this minimum, the particle pressure is π­(*n*_min_) = 0. Furthermore, for the sake of simplicity, we now choose a high-temperature limit, θ_*i*_ ≫ θ_0_, valid for near-ambient conditions. Under this approximation, the expression for the internal energy eq 68 is significantly simplified, and yields

71 $$u_{i}=\Psi \left(n_{i}\right)+C_{V,i}\theta_{i}$$

Equation 71 can explicitly be inverted to express θ_*i*_ as a function of *u*_*i*_ and *n*_*i*_

72 $$\theta_{i}=\frac{u_{i}-\Psi \left(n_{i}\right)}{C_{V,i}}$$

Equations 62 and 72 represent the closure relations that define the particle LTh, which complement the GenDPDE EoM. These EoS are herein expressed in terms of dressed variables, i.e., in the energetic representation, for convenience. However, whenever the bare intensive variables are needed instead, such as in eq 60, eqs 16 and 17 can be used to transform to the entropic representation with no ambiguity.

### 3.3 Computational Details

All tests were performed using a Fortran code parallelized with OpenMP, developed in our group. The EoM eqs 46 and 47 were integrated numerically using a Velocity-Verlet scheme, whereas eq 48 was integrated using an Euler scheme, with an energy drift correction. The latter, evaluates the numerical energy drift at every time step and reestablishes the balance using the internal energy of the particles.

Simulations were performed in the microcanonical ensemble (constant total energy) using reduced units, determined as follows. For a given physical substance under consideration, we choose a reference thermodynamic state point, characterized by a temperature *T*_0_, pressure *P*_0_ and mass density ${\mathrm{\rho ̃}}_{0}$ . The physical particle number density is thus given by

73 $$\rho_{0}={\mathrm{\rho ̃}}_{0}\frac{N_{A}}{M_{w}}$$

where *N*_A_ is Avogadro’s number and $M_{w}$ is the molecular weight of the considered substance [kg/mol]. Assuming a CG level ϕ = 5, i.e., that each mesoparticle contains 5 physical particles, the mesoscopic particle number density becomes

74 $$n_{0}=\frac{\rho_{0}}{\phi }$$

Therefore, if the mass of the physical constituent is denoted by *m̃*, the mesoparticle mass is

75 $$m_{i}=\phi \mathrm{m̃}$$

The heat capacity of the mesoparticle *C*_V,*i*_ is thus related to that of the substance ${\mathrm{C̃}}_{V}$ [J/K·kg] by the relation

76 $$C_{V,i}={\mathrm{C̃}}_{V}\phi \mathrm{m̃}$$

Notice that the particle mass as well as the particle heat capacity increase proportionally to ϕ. Hence, the thermal velocity of a mesoparticle decreases with the degree of CG, as $v_{thermal}∼\sqrt{k_{B}T/m}∼\sqrt{k_{B}T/\left(\mathrm{m̃}\phi \right)}∼\phi^{-1/2}$ , cf. eq 75. In the same way, the particle internal energy fluctuations scale as $\delta u_{thermal}∼\sqrt{k_{B}C_{V}T^{2}}$ , so that the fluctuations in the particle temperature $\delta \theta_{thermal}∼\delta u_{thermal}/C_{V}∼1/\sqrt{\phi }$ , according to eq 76 (see also ref 30). Therefore, a choice of a small degree of CG ϕ = 5 aims at enhancing the mesoscopic nature of the system.

We now introduce the dimensionless units by defining the characteristic scales from the mesoscopic perspective. The unit of length is defined from the local mesoparticle density, according to

77 $$L_{ref}={\left(\frac{1}{n_{0}}\right)}^{1/3}$$

implying that the dimensionless reference number density is equal to 1, *by construction*. The unit of energy is set as

78 $$u_{ref}=\frac{L_{ref}^{3}}{\kappa_{T}^{0}}=\frac{1}{n_{0}\kappa_{T}^{0}}$$

Finally, the unit of mass is determined by the degree of CG

79 $$m_{ref}=\phi \frac{M_{w}}{N_{A}}$$

The unit of time then becomes

80 $$t_{ref}=\sqrt{\frac{m_{ref}L_{ref}^{2}}{u_{ref}}}$$

Hence, in reduced units, *R*_cut_^*^≡*R*_cut_/*L*_ref_, *n*_i_^*^≡*n*_*i*_ *L*_ref_^3^, *u*_i_^*^≡*u*_*i*_/*u*_ref_, θ_i_^*^≡*k*_B_θ_*i*_/*u*_ref_, π_i_^*^≡π_*i*_ *L*_ref_^3^/*u*_ref_≡π_*i*_ *κ*_*T*_^0^, *C*_*V*,i_^*^≡*C*_*V*,*i*_/*k*_B_, *B*_T_^*^≡*B*_*T*_ *κ*_*T*_^0^, *m*_i_^*^≡*m*_*i*_/*m*_ref_, and t* ≡ *t*/*t*_ref_.

Notice that, although typically *k*_B_ *T* = 1 in DPD simulations, in this work we choose the particle compressibility, κ_*T*_, as derived from the LTh pressure EoS (cf. eq 64), to center the energy scale in the potential interactions between particles, as it is customary in MD simulations. Indeed, in the latter with Lennard-Jones fluids, for example, the energy scale is taken to be ε, instead of *k*_B_ *T*. However, relations can be established to convert the numerical value of variables from one convention to the other. Effectively, any physical variable *y* will scale with the three independent units chosen, as [*y*] = *L* ^α^ *m* ^β^ *u* ^γ^. Thus, using (*) to indicate dimensionless variables in our convention, we have that *y* ^*^ = *y*/(*L*_ref_^*α*^ *m*_ref_^β^ *u*_ref_^γ^), according to eqs 77–79. Instead, using (**) to denote the standard convention (as in ref^9^), $y^{**}=yc_{0}^{\alpha /3}/m_{ref}^{\beta }{\left(k_{B}T\right)}^{\gamma }$ . Hence, the transformation rules are given by $y^{**}=y^{*}\phi^{\alpha /3}{\left(k_{B}T\kappa_{T}n_{0}\right)}^{\gamma }=y^{*}\phi^{\alpha /3}{\left(T^{*}\right)}^{\gamma }$ . This expression shows, e.g., that κ** = ϕT*. Notice that we have assumed that in the standard convention, the unit of length is obtained from the physical number density as 1/*c*_0_^1/3^, although other choices may use *R*_cut_ and hence the relation would accordingly change.

Equilibrium simulations have been performed in a cubic box with periodic boundary conditions (PBC) in all directions and containing *N* = 27,000 mesoparticles. After an equilibration period of 1000 time units, in which the velocity is rescaled to lead the system toward the desired nominal temperature, a production run of 2000 time units has been performed in all tests. The time step size and, consequently, the total number of iterations required to complete the simulations were adjusted to ensure satisfactory energy conservation in all cases. To test this point, we have checked that the difference between the average kinetic temperature and the particle temperature is less than 0.1%, indicating that the effect of the energy drift correction is negligible during the simulation.

The normalized quadratic weighting function eq 20 was used for calculating of the local density, whereas a non-normalized weighting function

81 $$\omega^{p,u}\left(r\right)={\left(1-\frac{r}{R_{cut}^{p,u}}\right)}^{2}$$

was used for the dynamic properties γ_*ij*_ and κ_*ij*_. We have furthermore chosen to set *R*_cut_ = *R*_cut_^*p*^ = *R*_cut_^*u*^ to introduce one single length scale at the mesoparticle level, for simplicity.

The mesoscopic friction coefficient, γ, and thermal conductivity, κ, have been estimated from their macroscopic counterparts using the approximated theoretical predictions^35–37^

82 $$\nu =\frac{45mk_{B}T_{0}}{4\pi \gamma R_{cut}^{3}}+\frac{2\pi \gamma R_{cut}^{5}n_{0}^{2}}{1575}$$

83 $$\lambda =\frac{45C_{V}k_{B}T_{0}}{2\pi \gamma R_{cut}^{3}}+\frac{2\pi \kappa R_{cut}^{5}n_{0}^{2}}{315T_{0}^{2}}$$

with ν, the experimental kinematic viscosity of the substance and λ, its thermal conductivity.^38^

## 4 Results and Discussion

In this section, we present and discuss the outcome of our numerical simulations, which consider liquid argon and water as test substances. By setting the local particle thermodynamic parameters in accordance with the physical state that we intend to reproduce (see below) for both the argon and water cases, we expect the mesoscopic model to replicate the behavior of the physical fluids at the reference state, characterized by a given temperature *T* = *T*_0_ and a mass density $\mathrm{\rho ̃}={\mathrm{\rho ̃}}_{0}$ . Attention is directed toward evaluating the equilibrium properties, with particular emphasis on the accurate determination of the particle volume, as a means to reproduce the experimental pressure and eliminate artifacts from density-dependent CG models.

Through an analysis of the results in terms of both the thermodynamic quantities and the local structure of the system, we demonstrate the fundamental role of eq 42 in accurately reproducing the liquid phase behavior. We show that under the selected conditions, the traditional local density estimator eq 18 introduces numerical artifacts, like the particle clustering that adversely affect the system pressure, which lead to a final state of the system far from the nominal conditions desired. Furthermore, we also examine the influence of the cutoff distance, *R*_cut_, on the calculated properties, as well as on its relationship with the scaling factor, *f*_cut_, in the case of eq 42. Lastly, we assess the differences in computational cost associated with the two density estimators by comparing the total simulation time required to complete the tests.

### 4.1 Argon

GenDPDE simulations were performed to describe liquid argon around a reference state defined by *T*_0_ = 125.7 K, *P*_0_ = 85.31 MPa and ${\mathrm{\rho ̃}}_{0}=1419.7kg/m^{3}$ . Under these conditions, the isothermal compressibility is κ_T_^0^ = 1.49 × 10^–9^ Pa^–1^, and the constant-volume heat capacity is ${\mathrm{C̃}}_{V}=5.20\times 10^{2}J/\left(kg\cdot K\right)$ .^38^ Using the parametrization protocol discussed in Section 3, the corresponding dimensionless parameters for the reference state are temperature *T*_0_^*^ = 0.0111, pressure *P*_0_^*^ = 0.127, and number density *n*_0_^*^ = 1.000 (by construction). The reference particle pressure is π_0_^*^ = 0.1161, and the constant-volume heat capacity is *C*_V_^*^ = 12.51. Moreover, the parameter γ_*T*_ appearing in the Tait EoS, eqs 62 and 72, has been set to 6.0 to best reproduce the EoS for the system.

As a dynamic method for mesoscale simulations, GenDPDE requires setting values for the dissipative coefficients, to complete the definition of the algorithm, even though the thermodynamic properties are insensitive to these. We thus choose a parametrization that produces close values for the viscosity and thermal conductivity of the system, by completeness. Hence, given that, at the selected state point, the kinematic viscosity of argon is ν = 1.69 × 10^–7^ m^2^/s and its thermal conductivity is λ = 0.139 W/(m ·K),^38^ we estimate the corresponding mesoscopic dynamic coefficients from eqs 82 and 83. In Table 1, the dimensionless γ* and κ* are reported as a function of the different values of *R*_cut_^*^, while ensuring the physical viscosity and thermal conductivity remain fixed.

**Table 1:** Dimensionless Mesoscopic Dynamics Coefficients for Liquid Argon as a Function of the Cutoff Distance^a^

*R*_cut_^*^	γ*	κ*
1.3365	23.42	0.0079
1.6839	7.37	0.0025
2.1564	2.14	0.00072

a The dissipative parameter values decrease with increasing *R*_cut_ to ensure that the viscosity and thermal conductivity remain fixed.

The time step, δt*, the corresponding number of iterations for the equilibration period, *N*_eq_, and the production run, *N*_pr_, as well as the total number of iterations, *N*_tot_, are also given as a function *R*_cut_^*^ in Table 2.

**Table 2:** Time Step and Number of Iterations for Liquid Argon Simulations as a Function of the Cutoff Distance

*R*_cut_^*^	δt*	*N*_eq_	*N*_pr_	*N*_tot_
1.3365	5 × 10^–4^	2 × 10^6^	4 × 10^6^	6 × 10^6^
1.6839	1 × 10^–3^	1 × 10^6^	2 × 10^6^	3 × 10^6^
2.1564	1 × 10^–3^	1 × 10^6^	2 × 10^6^	3 × 10^6^

Two computational experiment series are conducted using, on the one hand, the traditional local density estimator (LDE), given by eq 18, and, on the other hand, the proposed alternative, given by eq 42. These local density estimators are hereafter referred to as LDE-A and LDE-B, respectively. All simulation parameters listed above are common to both versions. In addition, for LDE-B, the scaling factor *f*_cut_ has also been tuned as a function of the cutoff distance, as specified in Table 3 below.

**Table 3:** Scaling Factor for Liquid Argon Simulations as a Function of the Cutoff Distance

*R*_cut_^*^	*f*_cut_
1.3365	1.41
1.6839	1.35
2.1564	1.33

Ensemble averages of the thermodynamic quantities (i.e., the dimensionless temperature, T*, density ${\mathrm{n̅}}^{*}$ , and pressure P*) were collected for liquid argon using both estimators, and are reported in Table 4 for all cutoff distances under consideration. In particular, the system pressure has been obtained as an average over instantaneous values calculated using the Virial formula

84 $$P\equiv \frac{1}{3V}\left\langle \sum_{i}\frac{p_{i}^{2}}{m_{i}}+\sum_{i}\sum_{j<i}r_{ij}\cdot f_{ij}^{C}\right\rangle$$

**Table 4:** Average Thermodynamic Quantities Obtained from Liquid Argon Simulations at Various Cut-Off Distances^a^

*R*_cut_^*^	T*		${\mathrm{n̅}}^{*}$		P*	
	LDE-A	LDE-B	LDE-A	LDE-B	LDE-A	LDE-B
1.3365	0.0115 ± 0.0001	0.0112 ± 0.0001	0.799 ± 0.001	0.997 ± 0.001	0.008 ± 0.001	0.136 ± 0.001
1.6839	0.0112 ± 0.0001	0.0112 ± 0.0001	0.811 ± 0.001	1.000 ± 0.001	0.011 ± 0.001	0.128 ± 0.001
2.1564	0.0111 ± 0.0001	0.0111 ± 0.0001	0.870 ± 0.001	1.000 ± 0.001	0.041 ± 0.001	0.127 ± 0.001

a Comparison of LDE-A and LDE-B highlights the differences in thermodynamic behavior resulting from the local density definitions given by eqs. 18 and 42, respectively.

For both LDEs, the average temperature is satisfactorily close to the nominal value *T*_0_^*^ for all values of *R*_cut_^*^ (cf. Table 4). However, the density calculated with LDE-A is consistently underestimated and far from *n*_0_^*^. This is also reflected in the pressure, which is significantly lower than *P*_0_^*^ for the LDE-A measurements, irrespective of the cutoff distance considered. Conversely, the use of eq 42 in LDE-B enables the proper estimation of ${\mathrm{n̅}}^{*}$ through the tuning of *f*_cut_. The resulting pressure provides a closer match to the expected nominal value. At this point, we should mention that, by increasing *R*_cut_, the repulsive force between particle pairs becomes weaker, due to the reduction in the local density fluctuations as the number of neighbors increases. The effect of this weakening is seen in the plots of the radial distribution function (see Figure 3): the profiles are smoother as *R*_cut_ increases. As a consequence, the width of the correlation hole is affected by the value of *R*_cut_ and, thus, the parameter *f*_cut_ is sensitive to the strength of the interaction forces (see Table 3). Therefore, the value of *f*_cut_ has to be reconsidered if different states with significantly different local structures are to be considered. Hence, our alternative density estimator is actually linked to the physical state that we aim at describing.

**Figure 3:**
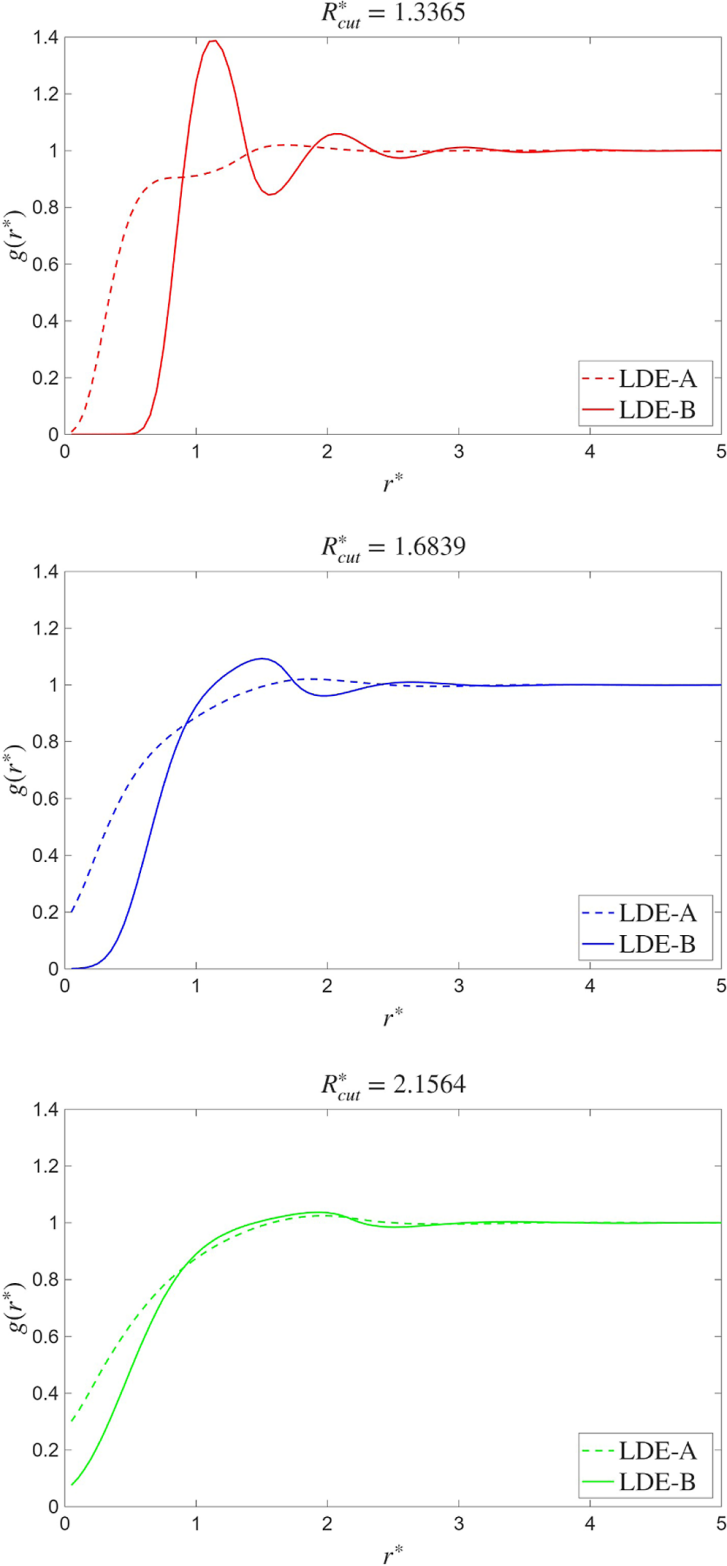
Argon radial distribution functions for *R*_cut_^*^ = 1.3365 (top), *R*_cut_^*^ = 1.6839 (middle), *R*_cut_^*^ = 2.1564 (bottom). Dashed lines refer to curves obtained using LDE-A, eq 18, whereas the continuous lines refer to LDE-B, eq 42.

Let us examine in more detail the thermodynamic behavior of the system in the case of LDE-A. The values assumed by ${\mathrm{n̅}}^{*}$ when considering eq 18 are systematically close to *n*_min_^*^ = 0.820, according to eq 70. As a consequence, the particle pressure π_i_^*^≃0, on average. Thus, following eq (B3), the system pressure is almost exclusively determined by the contribution due to the thermal agitation of the particles. For systems where the thermal agitation is dominant over the potential interactions, such as gases or supercritical fluids, the effect of the density underestimation is less relevant, as the correlation holes tend to be shallow. However, in liquid systems where potential interactions prevail, the correct estimation of the particle volume is crucial.

Inspection of the local structure of the system provides further insight on the nature of density-dependent potentials and the unphysical behavior caused by the use of LDE-A, eq 18. In Figure 3, the radial distribution function, *g*(*r*), is plotted for the different values of *R*_cut_^*^, and the results obtained with LDE-A and LDE-B are compared. Starting with the case *R*_cut_^*^ = 1.3365, we observe significantly different behaviors between the two LDEs. Notice that the strength of the repulsion forces in density-dependent potentials depends on the cutoff distance. Hence, the smaller the cutoff radius, the rougher the density variations that the central particle encounters in its motion as it explores a volume $V∼R_{cut}^{2}v\delta t$ , where v is the particle velocity. These density fluctuations δ*n* in this displacement are approximately given by $\delta n∼\pm \sqrt{\frac{\mathrm{n̅}}{v}}∼\frac{1}{R_{cut}}$ (using Poisson distribution for the estimate). Therefore, for small cutoffs, the density variations and interparticle forces are larger than for cases in which *R*_cut_ → ∞ and δ*n* → 0, as the particle always senses the average density. Then, it is precisely for the shortest cutoff (*R*_cut_^*^ = 1.3365) where the particle association issue is expected to be more noticeable. Effectively, in the case of LDE-A, particles are allowed to spuriously rearrange in order to accommodate the energetically more convenient, vanishing-pressure configuration. The *shoulder* observed in the *g*(*r*) from the LDE-A simulation at *r* ≃ 0.7 is a signature of such local clustering. Remarkably, the use of eq 42 in LDE-B allows us to suppress the clustering at distances *r* < 1. Notice that, in Figure 3 none of the radial distribution functions keep any connection to their physical equivalent, experimentally measurable, as the former represent the behavior of mesoscopic objects, not molecules, ad-hoc constructed to display given properties. Thus, there is no physical meaning that can be attributed to the differences between LDE-A and LDE-B in Figure 3, except for the absence of substructure at *r* < 1 in LDE-B. This is the feature expected by a physically consistent mesoscopic model. The LDE-A estimator, on the other hand, produces a spurious substructure which invalidates its application to liquid phases, as it eventually makes the system drift toward undesired thermodynamic states.

The behavior of the system in LDE-A tests is reminiscent of the so-called *pairing instability*, an infamous numerical issue discussed in the field of SPH.^39–42^ The pairing instability in SPH is an analogous clustering artifact also produced by the density estimator, which is defined through the same expression given in eq 18, using a variety of different kernel forms as weighting functions. The instability is observed in the form of particles that clump together to create closely paired structures and remain close during their motion for a significantly long time. In this context it is also qualitatively recognized that these pairings are a consequence of the formation of a more energetically favorable configuration, albeit undesired.^42^ We have clearly demonstrated such a correspondence by showing that the local density selected by the system is close to the minimum of the pressure potential Ψ­(*n*_*i*_) for the GenDPDE model discussed here (see eq 70 and Table 4). At the minimum, as π ≃ 0, any variation of the local density induces either a positive or negative pressure leading the density back to that at the minimum. In SPH, remedies to this issue have been proposed, based on computationally expensive Voronoi tessellation techniques,^40^ particle shifting,^43,44^ as well as Wendland kernels as weighting functions. The success of the latter has been argued to stem from having a non-negative Fourier transform.^42^ Notably, our weighting function eq 20 also shares this property, as its Fourier transform is written as

85 $$\begin{array}{ll} w\left(k\right)=30\left(\frac{4kr_{c}-6\;\sin kr_{c}+2kr_{c}\;\cos kr_{c}}{{\left(kr_{c}\right)}^{5}}\right) & >0,\\ & \forall k>0 \end{array}$$

However, the clustering still occurs in our simulations indicating that the spurious aggregation is more complex than a pairwise kernel-dependent effect. Rather, it is the consequence of the use of inhomogeneous weighting functions, which favor certain rearrangements that minimize the associated force potential through a poorly estimated particle volume. We have discarded the use of other kernels used in DPD, like Lucy’s,^45^ because its Fourier transform does not satisfy the condition ω­(*k*) ≥ 0, ∀*k* and it could mask the true origin of the pairing effect. The improvement presented in this article actually tends to correct the inhomogeneous nature of the weighting function, and as a consequence largely suppresses the formation of spurious cramming at distances *r* < 1.

Although in the present article we have limited the analysis to the weighting function eq 20, the impact of different weighting functions, such as Wendland kernels, on the density calculation in GenDPDE as well as SPH will be addressed elsewhere.

### 4.2 Water

Water simulations have been performed at near standard conditions, considering *T*_0_ = 293.16 K, *P*_0_ = 0.10 MPa and ${\mathrm{\rho ̃}}_{0}=998.20kg/m^{3}$ . The corresponding isothermal compressibility is κ_T_^0^ = 4.60 × 10^–10^ Pa^–1^, which is approximately 1 order of magnitude smaller than in argon at the conditions studied. The constant-volume heat capacity is ${\mathrm{C̃}}_{V}=4.15\times 10^{3}J/\left(kg\cdot K\right)$ .^38^ In dimensionless variables, the state point corresponds to a temperature *T*_0_^*^ = 0.0124, pressure *P*_0_^*^ = 4.6 × 10^–5^, and number density *n*_0_^*^ = 1.000 (by construction). Note that, while the order of magnitude of *T*_0_^*^ is the same in water and argon, the dimensionless pressure *P*_0_^*^ for water is 4 orders of magnitude smaller. From a physical standpoint, this is a signature of the peculiar nature of water as a strongly hydrogen-bonded liquid. This strong bonding between water molecules results in a rather high boiling temperature, but also a very low compressibility. As a consequence, the ideal-gas contribution of the mesoparticles to the system pressure, *T*_0_^*^ = 0.0124, is larger than the pressure for the state point considered, *P*_0_^*^ = 4.6 × 10^–5^, which results in a negative value for the reference particle pressure, π_0_^*^ = -0.0124 in the EoS eq 62. Furthermore, the constant-volume heat capacity is *C*_V_^*^ = 44.97, while the Tait EoS parameter γ_T_ has been set to 5.0.

The kinematic viscosity of water at ambient conditions is ν = 1.00 × 10^–6^ m^2^/s and its thermal conductivity is λ = 0.598 W/(m·K).^38^ The corresponding mesoscopic dynamics coefficients have again been obtained using eqs 82 and 83, and are reported in dimensionless form as a function of *R*_cut_^*^ in Table 5.

**Table 5:** Dimensionless Mesoscopic Dynamics Coefficients for Water as a Function of the Cutoff Distance^a^

*R*_cut_^*^	γ*	κ*
1.3365	75.20	0.015
1.6839	23.68	0.0047
2.1564	6.88	0.0014
2.4286	3.79	0.00075

a The value of the dissipative parameters decreases with increasing *R*_cut_ to keep the viscosity and thermal conductivity constant.

The values of δt*, *N*_eq_, *N*_pr_ and *N*_tot_ are given as a function *R*_cut_^*^ in Table 6.

**Table 6:** Time Step and Number of Iterations for Water Simulations as a Function of the Cutoff Distance

*R*_cut_^*^	δt*	*N*_eq_	*N*_pr_	*N*_tot_
1.3365	5 × 10^–5^	2 × 10^7^	4 × 10^7^	6 × 10^7^
1.6839	1 × 10^–4^	1 × 10^7^	2 × 10^7^	3 × 10^7^
2.1564	1 × 10^–4^	1 × 10^7^	2 × 10^7^	3 × 10^7^
2.4286	1 × 10^–4^	1 × 10^7^	2 × 10^7^	3 × 10^7^

Lastly, the values of *f*_cut_ as a function of the cutoff radius are reported in Table 7.

**Table 7:** Scaling Factor for Water Simulations as a Function of the Cutoff Distance

*R*_cut_^*^	*f*_cut_
1.3365	1.00
1.6839	1.01
2.1564	1.18
2.4286	1.21

The state point for water is characterized by an almost vanishing nominal pressure *P*_0_^*^ ∼ 0. In view of the discussion of Section 4.1, the nominal density *n*_0_^*^ = 1 lies very close to the value that minimizes the potential *n*_min_^*^ = 1.012 of the particle free energy eq 69. Our state point is thus in the vicinity of the conditions that give rise to the local aggregation artifact due to the convexity of Ψ­(*n*) around the minimum. The consequences of this situation are visible in terms of thermodynamic quantities in Table 8. As expected from the above considerations, both definitions of the local density estimators LDE-A, eq 18, and LDE-B, eq 42, return values that are close to the reference *n*_0_^*^. As such, the unphysical behavior of LDE-A that was evident in argon tests (cf. Table 4) is now concealed by the fact that *n*_0_^*^≃*n*_min_^*^. Pressure measurements also lie close to *P*_0_^*^, although they reveal that LDE-A overestimates *P*_0_^*^ when considering *R*_cut_^*^ = 1.3365, and underestimates it for *R*_cut_^*^ = 2.1564 and *R*_cut_^*^ = 2.4286. Only when *R*_cut_^*^ = 1.6839, *P* ^*^ ∼ *P*_0_^*^. Conversely, LDE-B overestimates the pressure for *R*_cut_^*^ = 1.3365 and *R*_cut_^*^ = 1.6839, whereas the values returned by *R*_cut_^*^ = 2.1564 and *R*_cut_^*^ = 2.4286 are reasonably close to *P*_0_^*^.

**Table 8:** Average Thermodynamic Quantities Obtained from Liquid Water Simulations at Various Cut-Off Distances^a^

*R*_cut_^*^	T*		${\mathrm{n̅}}^{*}$		P*	
	LDE-A	LDE-B	LDE-A	LDE-B	LDE-A	LDE-B
1.3365	0.0127 ± 0.0001	0.0126 ± 0.0001	1.000 ± 0.001	1.006 ± 0.001	0.0032 ± 0.0002	0.0099 ± 0.0001
1.6839	0.0125 ± 0.0001	0.0124 ± 0.0001	0.998 ± 0.001	1.001 ± 0.001	–0.0008 ± 0.0002	0.0028 ± 0.0002
2.1564	0.0124 ± 0.0001	0.0124 ± 0.0001	0.997 ± 0.001	1.000 ± 0.001	–0.0033 ± 0.0002	0.0008 ± 0.0001
2.4286	0.0125 ± 0.0001	0.0124 ± 0.0001	0.997 ± 0.001	1.000 ± 0.001	–0.0032 ± 0.0002	0.0001 ± 0.0001

a Comparison of LDE-A and LDE-B highlights the differences in thermodynamic behavior resulting from the local density definitions given by eqs 18 and 42, respectively.

Despite the thermodynamic properties of the system being relatively close to the nominal values, the fact is that eq 62 is actually outside its range of applicability when used to describe water. To analyze the reasons for this latter statement, we highlight the general weaknesses of density-dependent potentials when used to model CG fluids in condensed phases.

First, if the local density is close to the minimum of Ψ­(*n*_*i*_), pairing will occur as the particles locally rearrange to maintain this minimum energy condition, thus balancing attraction and repulsion. Thus, the dynamics of the system under thermal agitation produces spurious local structures in the form of short chain-like structures which we will refer to as filaments (see Figure 4). The spontaneous formation of these structures exploits an inhomogeneous weighting function for the evaluation of the local density (regardless of the nature of its Fourier transform). Second, the use of the LDE-B model for the evaluation of the particle volume introduces an improvement to the situation because the impact of the inhomogeneity of the kernel is strongly reduced by our new method. However, the change is only apparent, as the problem still remains because it is actually *intrinsic* to density-dependent potentials: many different particle configurations lead to the same value of the local density. As a consequence, the physical behavior of our CG fluid representing water deviates from what should be expected. This can be seen, e.g., by the shapes of the radial distribution functions, which in many cases of the LDE-A model do not decay to 1 at distances several times larger than *R*_cut_, suggesting that the system forms long-range structures (see Figure 5). Moreover, the value of *g*(*r*) near zero is nonvanishing (except for the LDE-B case with *R*_cut_^*^ = 1.3365), suggesting that every particle has some neighbors in its immediate vicinity, which is the signature of the clustering. In Figure 4, we provide clear evidence of the filamentous structures formed in the system for the LDE-A model. Therefore, the validity of an EoS of the type of eq 62 requires the particle interaction to be repulsive for all the ranges of density expected for the problem under study. Roughly speaking, the reliability of the model requires π_0_^*^>0 and sufficiently large to ensure that repulsions will keep the CG particles free from many-body attractions spuriously created by the local density estimation. Notice that the main difference between our top-down and bottom-up models is that our mesoparticle contains many physical molecules, rather than one single center representing one water molecule with CG properties (e.g., refs 46–48 ). From our results, it is apparent that at low levels of CG density-dependent potentials do not produce the expected results. Instead, other types of interactions, such as pairwise repulsions, should be included to compensate the strong associative attraction and thus prevent the formation of substructures that spoil the local density estimation and produce wrong values of the interparticle forces.

**Figure 4:**
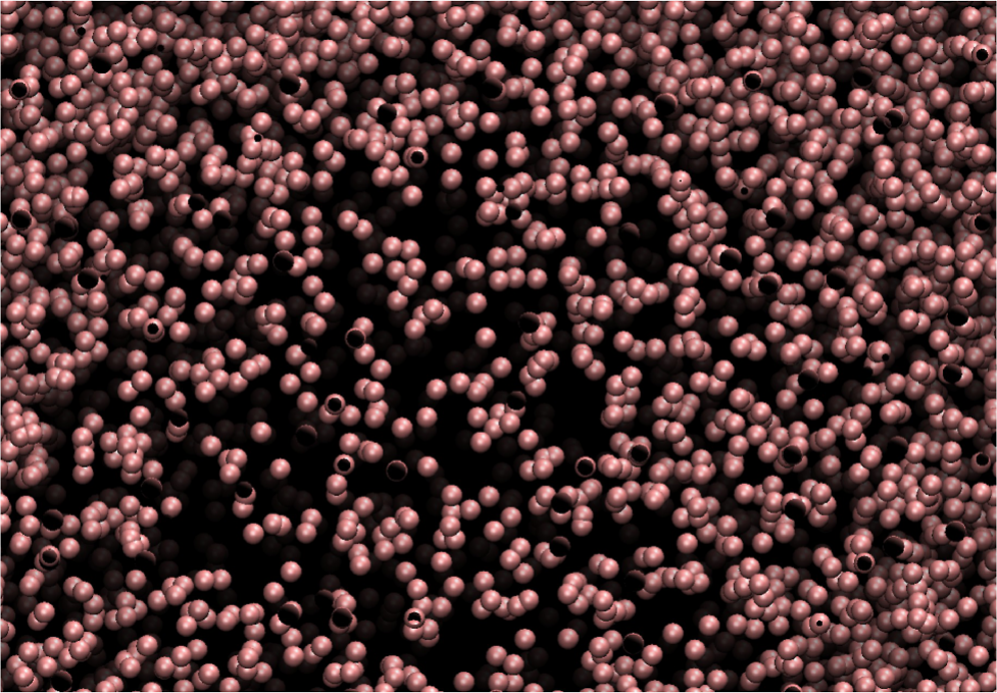
Chain structures and voids in the water model LDE-A at *R*_cut_ = 2.4286.

**Figure 5:**
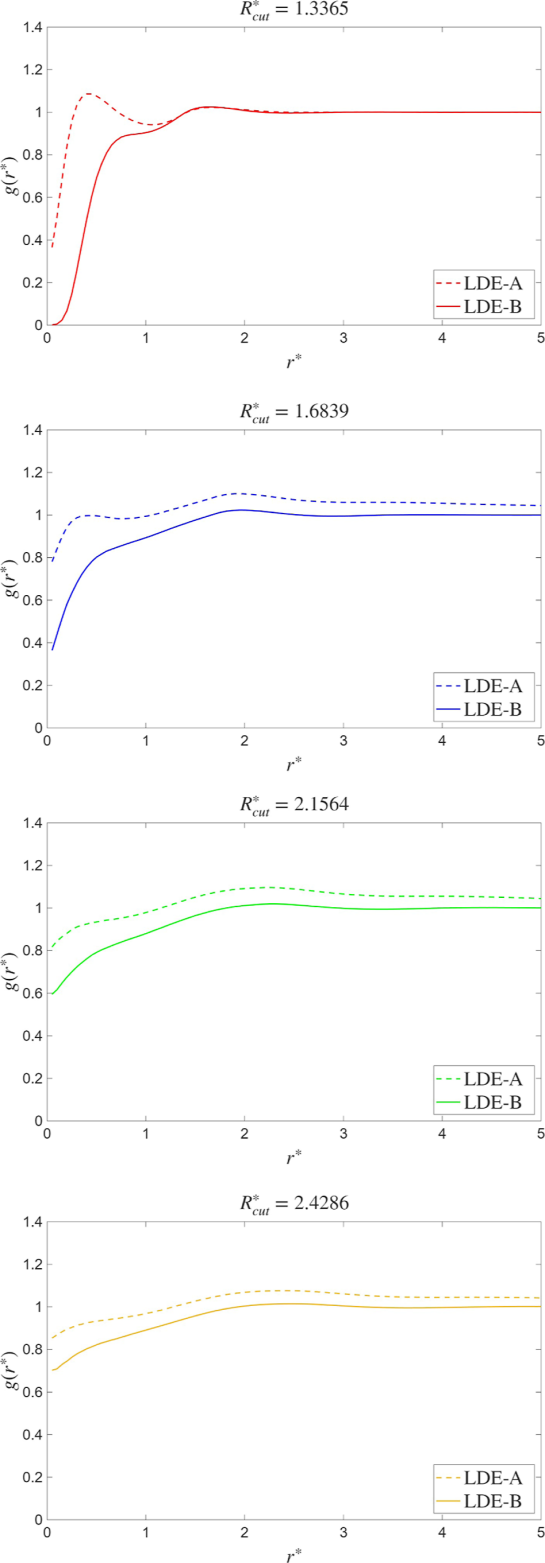
Water radial distribution functions for *R*_cut_^*^ = 1.3365 (top), *R*_cut_^*^ = 1.6839 (upper-middle), *R*_cut_^*^ = 2.1564 (lower-middle), *R*_cut_^*^ = 2.4286 (bottom). Dashed lines refer to curves obtained with LDE-A, eq 18, whereas the continuous lines refer to LDE-B, eq 42.

### 4.3 Computational Efficiency

For the liquid argon simulations, the measured wall-clock times per simulation time step are reported for the various cutoff distances in Table 9. By comparison of LDE-A and LDE-B simulation wall-clock times, we conclude that the LDE redefinition of eq 42 does not significantly affect the performance of the GenDPDE algorithm, thus preserving computational efficiency.

**Table 9:** Wall-Clock Time per Simulation Time Step for LDE-A and LDE-B Liquid Argon Simulations at Different Cut-Off Distances^a^

*R*_cut_^*^	time per time step [s]	
	LDE-A	LDE-B
1.3365	0.0151	0.0161
1.6839	0.0153	0.0185
2.1564	0.0190	0.0196

a All simulations were conducted using OpenMP parallelization with 16 threads on the same hardware.

## 5 Conclusions

In this work, we have investigated the application of the Generalized Dissipative Particle Dynamics with Energy conservation (GenDPDE) approach to the simulation of liquid phases, considering argon and water as paradigmatic test substances. In a previous analysis, in which phase separation was addressed as a case study,^24^ GenDPDE has been shown to slightly underestimate the coexistence densities, due to the use of the traditional local density estimator eq 18, a customary choice in many-body DPD calculations.^16,17,21^ In this article, we have demonstrated that the current use of inhomogeneous weighting functions, from which the particle volume is inferred, has a detrimental impact on the calculation of the properties of liquid systems. More specifically, we have focused our attention on the thermodynamic properties of the system, in terms of macroscopic system temperature, density and pressure, as well as on its local structure, the latter in terms of the radial distribution function. We have found that use of the traditional local density estimator allows the CG mesoparticles to aggregate locally into spurious configurations, formed to minimize the particle density-dependent potential. This is possible because, using the compressibility to define the scale of energy, the importance of the thermal agitation, i.e., the ideal-gas contribution to the pressure, is subdominant in liquids. Thus, the structure of the CG system is dominated by the many-body potential interactions. This undesired behavior is at the core of the pairing instability observed in the analogous particulate model Smoothed Particle Hydrodynamics, which is driven by a similar energy minimization mechanism. Although different solutions have already been proposed to eliminate the pairing instability in the context of SPH,^40,42–44^ we explored an alternative route within the GenDPDE framework, based on a redefinition of the local density estimator that yields a more accurate evaluation of the particle volume while also retaining modeling simplicity and computational efficiency. We have demonstrated that such redefinition allows us to recover the correct system behavior in terms of both, thermodynamic quantities and local structure, eliminating the aggregation artifact. This is particularly evident in the argon case, where, for relatively small *R*_cut_ and the use of the kernel eq 18, the measured local density lies close to the potential energy minimum and far from the expected nominal conditions. The system exploits the kernel inhomogeneity and the many-body nature of the local density determination, to create spurious structures visible in the *g*(*r*) (see Figure 3). Once the system is placed at the energy minimum, the interaction force eq 50 is virtually zero and can switch from repulsion to attraction by changes in the local density. When observed in the simulations, particles tend to move in small clusters in order to maintain the minimum energy condition, which gives rise to the aforementioned pairing instability in SPH.

The case of water, on the other hand, lies out of the previous considerations due to its intrinsic nature. Effectively, the low value of the nominal pressure *P*_0_^*^ ∼ 10^–5^, which is significantly smaller than the ideal-gas contribution of the mesoparticles ∼10^–2^, requires the dominant contribution of the interparticle force to be attractive, unlike in argon. Under this condition, we have seen that the local structures leading to pairing spontaneously occur for both the LDE-A and LDE-B models, i.e., independently of the local density estimator used. This effect is simply due to the fact that the functional relation between local particle configuration and local density is multivalued. Hence, the dominant attractive force tends to push the system toward the minimum of the potential energy, producing the strong density inhomogeneities observed in Figures 4 and 5. As a consequence, the fact that the thermodynamic properties are relatively well described by our EoS for water needs to be considered only as a coincidence, as the nominal density of the reference state is very close to the potential minimum.

Finally, note that as the split between kinetic and potential contributions to the total energy of the mesoparticle system depends on the level of CG, one could reduce the ideal gas contribution to the pressure by increasing ϕ. In our analysis, we have arbitrarily chosen ϕ = 5, but a choice ϕ ≥ 5000 would change the sign of the dominant pressure term and then the use of eq 62 would be legitimate under these conditions (standard SPH falls within the realm of a substantially large degrees of CGing). Thus, this perspective suggests that for mesoscale degrees of CGing, interparticle potentials must retain sufficient complexity to be capable of adequately describing the physical phenomena.

Setting aside ill-behaved cases, such as water under the CG conditions studied, the key improvement introduced by the redefined estimator eq 42 renders the GenDPDE method suitable for the qualitative and quantitative analysis of liquid phases at the mesoscopic level. This represents an important achievement in the context of DPD-like models, where the simulation of liquids with the aim of producing quantitative results has proven to be challenging. Although the analysis has been carried out for one-component systems, the application to multicomponent systems is straightforward. Actually, the particle volume entering the LTh description is determined by the space left by the surrounding particles irrespective of their chemical nature. This fact does not exclude the possibility that the model contains other contributions that are dependent on the given species which will have their specific form, as it would be the case in charged systems. On the other hand, for heterogeneous systems, like liquids near interfaces, CG potentials cannot rely only on the local density (particle volume) but they should include additional contributions describing the CG interfacial properties, apart from the volume term. All these considerations lie beyond the scope of the present article and will be addressed elsewhere. Finally, due to the capacity of GenDPDE to simulate scenarios involving heat flux, the analysis could be extended to, e.g., situations in which thermal gradients are present and other nonequilibrium situations. Thus, phenomena that are relevant for both academic and industrial applications, such as the thermophoresis in colloidal suspensions, material separation, among many others, could potentially be addressed, and comparisons with experimental findings could be drawn.

## Derivation of the Expression for the Particle Volume

With the aim of rigorously deriving the expression for the particle volume given in eq 42, let us consider again the definition eq 24

A1 $$V_{i}\equiv \int \Delta r\varphi_{i}^{*}\left(\Delta r\right)$$

where

A2 $$\varphi_{i}\left(\Delta r\right)≃\frac{\mathrm{w̃}\left(|\Delta r|\right)}{\mathrm{w̃}\left(|\Delta r|\right)+n_{i}^{b}}$$

with *n*_*i*_^*b*^ introduced in eq 18. We also consider a reduced cutoff distance ${\mathrm{R̃}}_{cut}\equiv R_{cut}/f_{cut}$ for the quadratic weighting function *w*(|Δ**r**|), reading

A3 $$\mathrm{w̃}\left(|\Delta r|\right)=\frac{15}{2\pi {\mathrm{R̃}}_{cut}^{3}}{\left(1-\frac{\left|\Delta r\right|}{{\mathrm{R̃}}_{cut}}\right)}^{2}$$

A suitable choice of *f*_cut_ ensures that the particle volume is accurately calculated. With these definitions, the integral in eq [Disp-formula fdA1] can be evaluated analytically. We begin by substituting eq [Disp-formula fdA2] with [Disp-formula fdA3] into eq [Disp-formula fdA1], so that

A4 $$\begin{array}{ll} V_{i} & =\int d\Delta r\frac{\mathrm{w̃}\left(|\Delta r|\right)}{\mathrm{w̃}\left(|\Delta r|\right)+n_{i}^{b}}\\ & =4\pi \int d\Delta r\Delta r^{2}\frac{\mathrm{w̃}\left(\Delta r\right)}{\mathrm{w̃}\left(\Delta r\right)+n_{i}^{b}}\\ & =4\pi \int_{0}^{{\mathrm{R̃}}_{cut}}d\Delta r\Delta r^{2}\frac{{\left(1-\frac{\Delta r}{{\mathrm{R̃}}_{cut}}\right)}^{2}}{{\left(1-\frac{\Delta r}{{\mathrm{R̃}}_{cut}}\right)}^{2}+k_{i}} \end{array}$$

with Δ*r* = |Δ**r**| and $k_{i}=\left(2\pi /15\right){\mathrm{R̃}}_{cut}^{3}n_{i}^{b}$ . Eq [Disp-formula fdA4] is reformulated by introducing the dimensionless variable $x=\Delta r/{\mathrm{R̃}}_{cut}$ , yielding

A5 $$\begin{array}{ll} V_{i} & =4\pi {\mathrm{R̃}}_{cut}^{3}\int_{0}^{1}dxx^{2}\frac{{\left(1-x\right)}^{2}}{{\left(1-x\right)}^{2}+k_{i}}\\ & =\frac{4\pi }{3}{\mathrm{R̃}}_{cut}^{3}-\int_{0}^{1}dxx^{2}\frac{k_{i}}{{\left(1-x\right)}^{2}+k_{i}} \end{array}$$

Applying a further change of variable by defining $z=\left(1-x\right)/\sqrt{k_{i}}$ , we rewrite eq [Disp-formula fdA5] as

A6 $$V_{i}=\frac{4\pi }{3}{\mathrm{R̃}}_{cut}^{3}-4\pi {\mathrm{R̃}}_{cut}^{3}\sqrt{k_{i}}\int_{0}^{1/\sqrt{k_{i}}}dz\frac{{\left(1-\sqrt{k_{i}}z\right)}^{2}}{z^{2}+1}$$

This final integral can be evaluated exactly. The resulting expression for the particle volume is

A7 $$\begin{array}{ll} V_{i}= & \frac{4\pi }{3}{\mathrm{R̃}}_{cut}^{3}-4\pi {\mathrm{R̃}}_{cut}^{3}\sqrt{k_{i}}\{\left(1-k_{i}\right)\arctan\left(\frac{1}{\sqrt{k_{i}}}\right)\\ & +\sqrt{k_{i}}\left[1-\ln\left(\frac{k_{i}+1}{k_{i}}\right)\right]\} \end{array}$$

which is the result reported in eq 42.

## The Excess Pressure

The macroscopic thermodynamic behavior of the mesoscopic particles in the canonical ensemble is given by^30^

B1 $$F=-k_{B}T\;\ln\left(Q_{N}\right)$$

where *F* is the Helmholtz free energy of the system, and

B2 $$Q_{N}=\frac{1}{h^{3N}\varepsilon^{N}N!}\int d\mathrm{\Gamma ̃}P_{eq}\left(\mathrm{\Gamma ̃}\right)$$

is the canonical partition function, with $P_{eq}\left(\mathrm{\Gamma ̃}\right)$ the equilibrium probability distribution defined in eq 2. In eq [Disp-formula fdB2], *h* is the Planck constant, ε is an energy scale representing the minimum separation between energy levels, and the factor *N*! accounts for particle indistinguishability. These prefactors make *Q*_*N*_ dimensionless. Equation [Disp-formula fdB1] provides all the information on the thermodynamics of the system from a macroscopic perspective and establishes a bridge between the mesoscopic LTh description and the macroscopic behavior. In particular, it allows us to derive the EoS for the overall pressure of the system, *P*. From^49^ we write

B3 $$\begin{array}{ll} P & ={-\frac{\partial F}{\partial V}|}_{T}=\\ & =k_{B}T\frac{N}{V}-\frac{T}{3V}\left\langle \sum_{i,j<i}\left(\frac{1}{n_{i}^{2}}\frac{{\mathrm{\pi ̃}}_{i}}{{\mathrm{\theta ̃}}_{i}}\zeta_{i}+\frac{1}{n_{j}^{2}}\frac{{\mathrm{\pi ̃}}_{j}}{{\mathrm{\theta ̃}}_{j}}\zeta_{j}\right)r_{ij}w^{\prime }\left(r_{ij}\right)\right\rangle =\\ & =k_{B}T\frac{N}{V}-\frac{1}{3V}\left\langle \sum_{i,j<i}\left(\frac{\pi_{i}}{n_{i}^{2}}\zeta_{i}+\frac{\pi_{j}}{n_{j}^{2}}\zeta_{j}\right)r_{ij}w^{\prime }\left(r_{ij}\right)\right\rangle =\\ & =k_{B}T\frac{N}{V}+P^{ex} \end{array}$$

where *V* is the system total volume. The right-hand side of eq [Disp-formula fdB3] can be expressed in terms of the instantaneous densities ρ̂(r), i.e.

B4 $$\begin{array}{ll} P^{ex} & =-\frac{T}{3V}\left\langle \sum_{i,j<i}\left(\frac{1}{n_{i}^{2}}\frac{{\mathrm{\pi ̃}}_{i}}{{\mathrm{\theta ̃}}_{i}}\zeta_{i}+\frac{1}{n_{j}^{2}}\frac{{\mathrm{\pi ̃}}_{j}}{{\mathrm{\theta ̃}}_{j}}\zeta_{j}\right)r_{ij}w^{\prime }\left(r_{ij}\right)\right\rangle ≃\\ & ≃-\frac{T}{3V}\frac{1}{2}\int dr\int dr^{\prime }\left\langle \left(\frac{1}{n^{2}\left(r\right)}\frac{\mathrm{\pi ̃}\left(r\right)}{\mathrm{\theta ̃}\left(r\right)}\zeta \left(r\right)+\frac{1}{n^{2}\left(r^{\prime }\right)}\frac{\mathrm{\pi ̃}\left(r^{\prime }\right)}{\mathrm{\theta ̃}\left(r^{\prime }\right)}\zeta \left(r^{\prime }\right)\right)\Delta rw^{\prime }\left(\Delta r\right)\mathrm{\rho ̂}\left(r\right)\mathrm{\rho ̂}\left(r^{\prime }\right)\right\rangle \end{array}$$

where Δ*r* ≡|**r** – **r**′|. In a homogeneous and isotropic system, the particle pressure and temperature are independent of the positions. The average of π̃/θ̃ over the energy fluctuations factorizes with respect to the position average involving the local densities. Then, using the definition of the pair distribution function, we can write

B5 $$P^{ex}≃-\frac{T}{3V}\frac{V}{2}\frac{\rho^{2}}{n^{2}}2\left\langle \frac{\mathrm{\pi ̃}}{\mathrm{\theta ̃}}\right\rangle \zeta \int d\Delta r\Delta rw^{\prime }\left(\Delta r\right)g\left(\Delta r\right)$$

where we have used the homogeneity condition to evaluate one of the volume integrals. If the particle interaction creates a significant correlation hole, the integration on the right-hand side cannot be developed further. However, if we can assume that the interaction is weak, then *g*(Δ*r*) ≃ 1 all over the integration domain. Then, we can integrate by parts the remaining term in this last equation, to obtain

B6 $$P^{ex}≃T\frac{\rho^{2}}{n^{2}}\left\langle \frac{\mathrm{\pi ̃}}{\mathrm{\theta ̃}}\right\rangle \zeta \left(4\pi \int_{0}^{R_{cut}}d\Delta r\Delta r^{2}w\left(\Delta r\right)\right)$$

The last term between brackets in this expression corresponds to the normalization integral for the weighting function, eq 19. Finally, in this limit, ζ ≃ 1 and *n* ≃ ρ, leading to the final expression

B7 $$P^{ex}≃T\left\langle \frac{\mathrm{\pi ̃}}{\mathrm{\theta ̃}}\right\rangle$$

as in eq 7. Applying the same procedure to the dressed pressure π, it is easy to obtain

B8 $$P^{ex}≃\left\langle \pi \right\rangle$$

which corresponds to eq 15.

## Notes

Disclaimer This work reflects only the authors’ view and the Agency is not responsible for any use that may be made of the information it contains. The views and conclusions contained in this document are those of the authors and should not be interpreted as representing the official policies, either expressed or implied, of the Army Research Office or the U.S. Government. The U.S. Government is authorized to reproduce and distribute reprints for Government purposes notwithstanding any copyright notation herein.
